# Likelihood-Based Gene Annotations for Gap Filling and Quality Assessment in Genome-Scale Metabolic Models

**DOI:** 10.1371/journal.pcbi.1003882

**Published:** 2014-10-16

**Authors:** Matthew N. Benedict, Michael B. Mundy, Christopher S. Henry, Nicholas Chia, Nathan D. Price

**Affiliations:** 1 Department of Chemical and Biomolecular Engineering, University of Illinois at Urbana-Champaign, Urbana, Illinois, United States of America; 2 Center for Individualized Medicine, Mayo Clinic, Rochester, Minnesota, United States of America; 3 Mathematics and Computer Science Division, Argonne National Laboratory, Lemont, Illinois, United States of America; 4 Department of Surgery, Mayo Clinic, Rochester, Minnesota, United States of America; 5 Department of Physiology and Bioengineering, Mayo Clinic, Rochester, Minnesota, United States of America; 6 Institute for Systems Biology, Seattle, Washington, United States of America; The Pennsylvania State University, United States of America

## Abstract

Genome-scale metabolic models provide a powerful means to harness information from genomes to deepen biological insights. With exponentially increasing sequencing capacity, there is an enormous need for automated reconstruction techniques that can provide more accurate models in a short time frame. Current methods for automated metabolic network reconstruction rely on gene and reaction annotations to build draft metabolic networks and algorithms to fill gaps in these networks. However, automated reconstruction is hampered by database inconsistencies, incorrect annotations, and gap filling largely without considering genomic information. Here we develop an approach for applying genomic information to predict alternative functions for genes and estimate their likelihoods from sequence homology. We show that computed likelihood values were significantly higher for annotations found in manually curated metabolic networks than those that were not. We then apply these alternative functional predictions to estimate reaction likelihoods, which are used in a new gap filling approach called *likelihood-based gap filling* to predict more genomically consistent solutions. To validate the likelihood-based gap filling approach, we applied it to models where essential pathways were removed, finding that likelihood-based gap filling identified more biologically relevant solutions than parsimony-based gap filling approaches. We also demonstrate that models gap filled using likelihood-based gap filling provide greater coverage and genomic consistency with metabolic gene functions compared to parsimony-based approaches. Interestingly, despite these findings, we found that likelihoods did not significantly affect consistency of gap filled models with Biolog and knockout lethality data. This indicates that the phenotype data alone cannot necessarily be used to discriminate between alternative solutions for gap filling and therefore, that the use of other information is necessary to obtain a more accurate network. All described workflows are implemented as part of the DOE Systems Biology Knowledgebase (KBase) and are publicly available via API or command-line web interface.

## Author Summary

Genome-scale metabolic modeling is a powerful approach that allows one to computationally simulate a variety of metabolic phenotypes. However, manually constructing accurate metabolic networks is extremely time intensive and it is thus desirable to have automated computational methods for providing high-quality metabolic networks. Incomplete knowledge of biological chemistries leads to missing, ambiguous, or inaccurate gene annotations, and thus gives rise to incomplete metabolic networks. Computational algorithms for filling these gaps in a metabolic model rely on network topology based approaches that can result in solutions that are inconsistent with existing genomic data. We developed an algorithm that directly incorporates genomic evidence into the decision-making process for gap filling reactions. This algorithm both maximizes the consistency of gap filled reactions with available genomic data and identifies candidate genes for gap filled reactions. The algorithm has been integrated into KBase's metabolic modeling service, an automated metabolic network reconstruction framework that includes the ModelSEED automated metabolic reconstruction tools.

## Introduction

Genome-scale metabolic models (GEMs) integrate available information about metabolism to provide a basis for holistic modeling and prediction of metabolic phenotypes [1]. GEMs have been utilized broadly [2], [3], [4], [5] across all three domains of life [6] to accelerate research in such areas as network evolution [7], [8], [9], synthetic biology [10], [11], and the discovery of novel drug targets [12]. However, achieving a sufficiently accurate metabolic model to enable high utility currently requires a very time-intensive manual reconstruction process, often taking many months or even years to complete [13]. As the throughput of sequencing technologies continues to increase and as research on microbial populations produces more and more genomes [14], there is a growing need for methods that automate high-quality metabolic model reconstruction.

Since the advent of genome-scale metabolic modeling, protocols [13], databases [15], [16], [17], algorithms [18], [19], [20] and toolboxes [20], [21], [22] have been developed to help systematize the lengthy and iterative process of collecting, curating, and integrating large volumes of biochemical knowledge. There have also been previous efforts to fully automate this process, including, notably, the Department of Energy's ModelSEED [20]. Despite these important advances, significant barriers to high-quality automated metabolic reconstructions still persist. Even with human curation, ambiguous or incorrect annotations are still pervasive [23]. Incomplete annotations leave gaps in the metabolic networks that need to be filled in order to make simulation possible [13], [24]. Inaccurate annotations also give rise to the need to identify and assess the merits of alternative annotations for genes, a process that typically done manually as part of the model curation process [13]. An automated approach to model building that accounts for alternative annotations would help expedite manual curation and ensure that the draft models maximally account for alternatives that can be identified based on available data.

Existing algorithms for filling gaps, or dead-end reactions, in metabolic networks broadly fall into approaches based on network topology [19], [25], pre-defined pathways [26], or phenotype data [27], [28], [29]. Parsimony-based algorithms such as GapFill identify dead-end reactions in a metabolic network and identify the minimum number of modifications to the network that can be made to activate those reactions [19]. Variations of GapFill have been developed that assign specific penalties based on thermodynamics or database incompleteness [25]. Pathway-based algorithms, such as that implemented in the PathwayTools [26], automatically complete pre-defined pathways that have sufficient representation in the draft model. Finally, several algorithms use phenotype data to help choose gap filling pathways, including OMNI, which maximizes model consistency with reaction rate data [27], GrowMatch, which maximizes consistency with experimental growth/no growth results [28], and MIRAGE, which maximizes the co-occurrence and co-expression of connected reactions [29]. Uniquely among these methods, MIRAGE also automatically identifies gene candidates for optimal gap fill solutions.

While existing methods capably activate the necessary reactions to allow growth simulations, they often do so by fitting to phenotype data. Such fitting may result in the inclusion of spurious pathways which cause failures when testing the models against independent datasets [30]. This is epitomized by a recent article that showed that in some cases, pruning these spurious pathways can lead to significant improvements in simulation accuracy [30]. Although genomic evidence may be incorporated after gap filling through human curation of potential solutions [31], these solutions are unlikely to fully reflect all the available knowledge of the genome. Methods that addresses both the resolution of dead-end metabolites and the identification of gene-reaction pairings for the reactions added to the model during the resolution of the gaps in the reaction network help researchers identify poorly-supported solutions when building models, thus helping to reduce over-fitting.

The goal of our work is to improve the quality of automatically generated metabolic reconstructions and models by explicitly incorporating alternative potential gene annotations and their estimated likelihoods into the gap filling process. We have developed a likelihood-based gap filling workflow that (1) assigns likelihood scores based on sequence homology to multiple annotations per gene and, from these, likelihoods for reactions in a network and (2) identifies maximum-likelihood pathways for gap filling using a mixed-integer linear programming (MILP) formulation. We have also developed a workflow to iteratively identify pathways that activates gene-associated orphaned reactions in a network and assesses the likelihood of these pathways. Critically, the likelihood-based approach makes the gap filling solution genome-specific and provides users with putative gene-protein-reaction relationships and confidence metrics for each result. We show that our likelihood-based approach improves the quantity and quality of new gene annotations compared to the existing gap filling algorithm. The resulting models have comparable accuracy when simulating high-throughput growth phenotype data, when compared with previous parsimony-based gap filling algorithms. The workflow tools are fully integrated within the Department of Energy's System Biology Knowledgebase (KBase), and are publicly available via both a web-based command line interface (available at http://kbase.us) and a web service API.

## Results

### Gap filling workflows using likelihood and parsimony-based approaches

Confidence scores are useful for building models and assessing the quality of the annotations, reactions and pathways therein [13]. We have developed a quantitative likelihood measurement for the evidence that a gene carries a specific annotated function and a technique by which these likelihood estimates can be converted into the likelihood of existence of a reaction in a cell's metabolic network (see **Methods**). Importantly, we simultaneously compute the likelihoods of multiple annotations for a single gene, which both broadens the space of testable annotation hypotheses in gap filling solutions and helps mitigate possible errors in the most likely annotation. We have also developed a method by which these annotation likelihoods are converted into likelihoods of metabolic reactions. These reaction likelihoods are useful to evaluate confidence in the inclusion of individual parts of a metabolic network.

**Figure 1:**
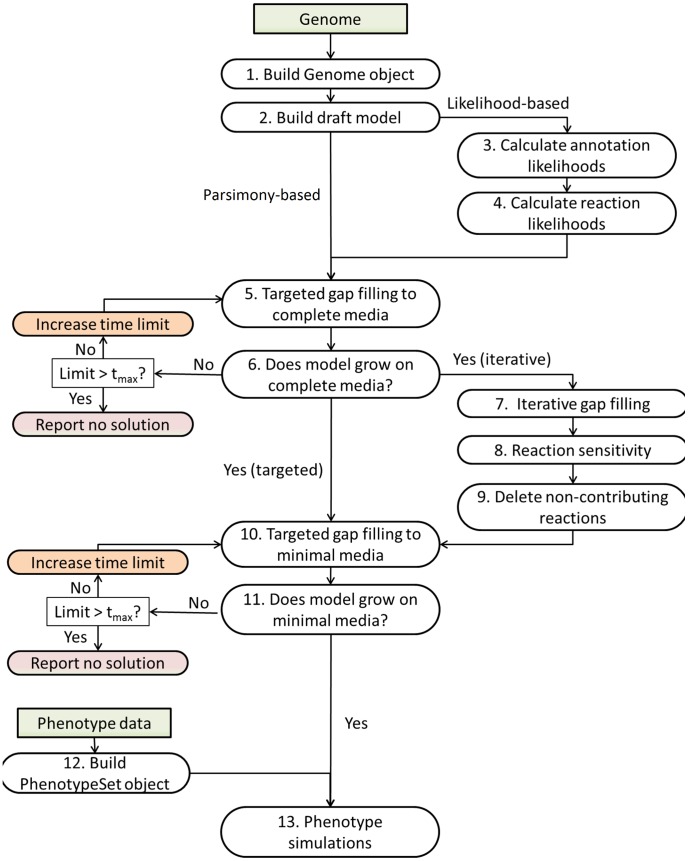
Gap filling workflows. We have developed four gap filling workflows and used them to generate the results in this paper: targeted parsimony-based gap filling, targeted likelihood-based gap filling, iterative parsimony-based gap filling, and iterative likelihood-based gap filling. The individual steps are described in detail in the methods, and the technical details of running them using the web interface are described in the supplementary material. Green boxes represent inputs to the workflows. “Limit” is the user-defined time limit and *t*_max_ is a system-defined maximum possible time limit for gap filling (currently one day) to prevent overloading the compute servers.

In order to assess the efficacy of the likelihood-based gap filling approach, we implemented four gap filling workflows (**Figure 1** and **Text S2**). These four workflows use two gap filling algorithms (parsimony-based versus likelihood-based gap filling) as applied to two separate gap filling strategies (targeted versus iterative gap filling). The goal of both algorithms is to alter the reaction network by adding new reactions or altering existing reactions. Here, we use the terms parsimony-based and likelihood-based to describe the two gap filling schemes according to their core mode of reaction addition. Both schemes can prioritize changes in reversibility or add special consideration to edge cases such as the addition of transporter reactions. However, in parsimony-based gap filling, the overall goal of parsimony-based gap fill is to make the least number of modifications in order to fill a gap. In general, this means that the shortest reaction path is incorporated into a network. In contrast, likelihood-based gap filling weights genomic evidence and takes into account how likely a reaction is to be included in the network. Likelihood-based gap filling favors reaction paths supported by evidence over paths without any supporting evidence from the genome.

In targeted gap filling, which is the most commonly used in the field, gap filling is used to activate one particular reaction in a model such as the biomass reaction [19]. The target reaction is activated either by adding new reactions to the model from a universal reaction database or by changing the reversibility of existing reactions in a model. A successful application of targeted gap filling enables simulations to be performed on the resulting model using an increasingly large suite of constraint-based analysis algorithms [32], [33].

In the second strategy, which we call *iterative gap filling*, targeted gap filling is applied iteratively to all inactive reactions in a network, hence maximizing the number of activated reactions in a network. High-priority reactions such as those in central metabolism are activated first (see Methods). One could imagine such an approach would sacrifice specificity for sensitivity (while targeted gap filling to achieve only biomass production would do the opposite). In the iterative gap filling workflows, a post-processing step is also used to reduce the redundancy resulting from attempting to activate every gene-associated reaction, to assess the value of each gap filled pathway in terms of how much of the original annotated network is corrected by the pathway, and optionally, to apply a cutoff to the cost of pathways added to the model (see **Methods** and **Test S2 and S3**).

### Likelihoods reflect a measure of confidence in predicted function

**Figure 2:**
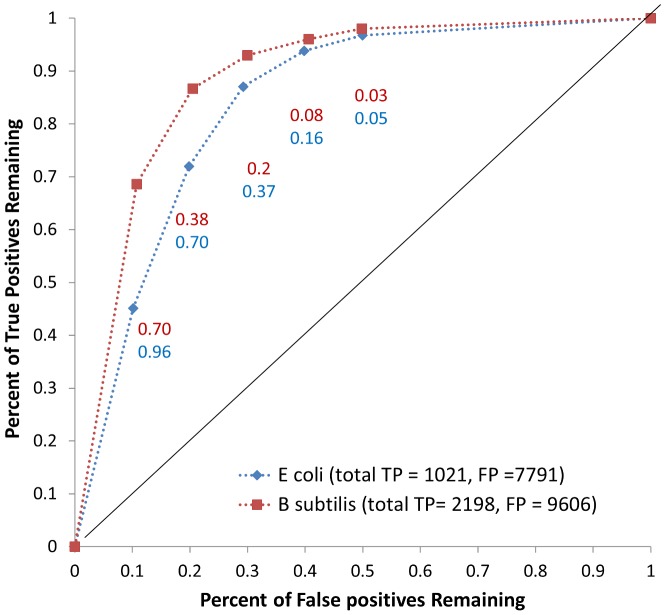
ROC curve for annotations. We computed the likelihood of all possible gene-reaction pairings from the ModelSEED database and compared the likelihoods of those pairings present in the iJR904 *E. coli* and iBSU1103 *B. subtilis* models (‘true positives’) to those which were not (‘false positives’). Each point in the curve represents the percentage of true and false positive linkages remaining at different likelihood cutoffs (labeled on each point). We found that there was a significant enrichment of true positives at high likelihood levels and false positives at low likelihood levels.

As part of the model curation process, it is necessary to evaluate the quality of each annotation and fix those which are found to be problematic [13]. We have implemented a simple method to estimate annotation likelihoods accounting for two sources of ambiguity: (1) sequence divergence between query genes and the genes in the reference database, and (2) inconsistencies in annotation within the reference database (see Methods). We characterized the utility of our reaction likelihoods by comparing the gene-reaction links created using our automated likelihood-based approach to those present in manually curated metabolic networks of *Escherichia coli* K12 [2] and *Bacillus subtilis* str. 168 [25] (note that iJR904, an older *E. coli* model than the most recent, was used because the ModelSEED database does not yet link annotations to periplasmic reactions and the gap filling implementation does not yet properly support compartmentalized models). We found that highly likely gene-reaction links were significantly enriched in the models compared to less-likely gene-reaction links (**Figure 2**) indicating that a higher likelihood score reflects higher confidence in the predicted function. We also identified large numbers of high-likelihood gene annotations that are not in the comparison models, which reflect promising candidates for further investigation and possible inclusion in the models.

### Proof of principle for likelihood-based gap filling

Unlike the parsimony-based gap filling approach, the likelihood-based approach is able to produce different solutions for different organisms, even if the starting network is identical, based on the organisms' genetic content. To demonstrate the utility of this approach in improving model quality, we identified a set of 32 reactions from the iBsu1103 genome-scale metabolic model of *B. subtilis* [25] that were predicted to be essential for growth and whose existence in the model was supported by literature evidence [34]. This set of reactions represented a gold standard set of reactions that should be incorporated into gap filling solutions if they were missing. We then removed all 32 gold standard reactions from the iBsu1103 model and applied the targeted parsimony-based gap filling and likelihood-based gap filling algorithms to restore biomass production in the knockout model.

In order to evaluate the effects of parameterizing each algorithm, we performed a sensitivity analysis on the effects of modifying penalties for adding transporters and for making thermodynamically unfavorable reversibility changes (see Supplemental Material). Larger penalties for these types of changes make it less favorable for the algorithm to pick them over other changes to the network when fixing network gaps. Since the removed set of reactions did not include transporters or reversibility changes, higher penalties for transporters or reversibility changes led to greater accuracy in the returned reactions. The penalty for transporters that maximized the accuracy of the returned pathways was higher for parsimony-based gap filling (55 or greater - equivalent to adding about 7 intracellular reactions on average) than for likelihood-based gap filling (25 or greater). The penalties for thermodynamically unfavorable reversibility changes that maximized accuracy were also higher for parsimony-based than likelihood-based gap fill (40 and 12, respectively). Therefore, likelihood-based gap filling reduced the need to have very large penalties for these categories of network changes in order to obtain accurate solutions.

**Figure 3:**
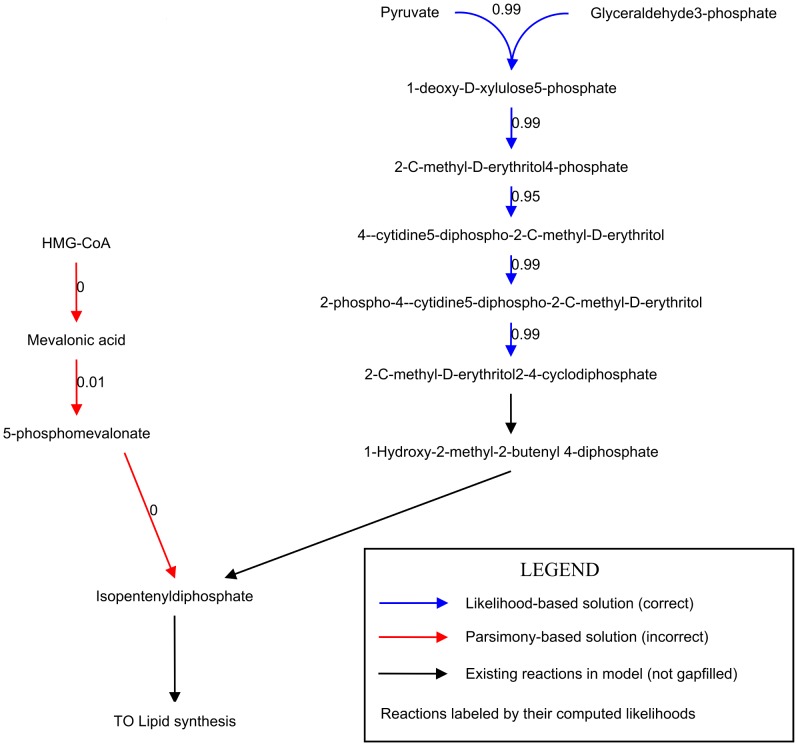
Proof of principle: Gap filling highly-likely reactions in *B. subtilis*. *B. subtilis* synthesizes lipids via the non-mevalonate pathway (blue) [37]. We removed this pathway from the *B. subtilis* genome-scale model and then tried to fill the gap using both the likelihood and parsimony-based approaches. The parsimony-based gap filling approach instead filled the gap with the mevalonate pathway (red), which is shorter but not supported by genetic evidence. The likelihood-based approach filled the gap with the correct pathway. Black indicates reactions that were not knocked out (there was no explicit link to literature evidence in the *B. subtilis* model). The numeric labels are the computed likelihoods of gap filling reactions.

Although both methods had the same number of tuning parameters available, likelihood-based gap filling successfully outperformed the parsimony-based method by replacing a maximum of 31 of the 32 gold-standard reactions. Parsimony-based gap filling only replaced only a maximum of 24 reactions, regardless of the chosen penalties (**Dataset S2**). The failures in parsimony-based gap filling were a result of picking shorter pathways to fill certain gaps for which longer pathways are the correct choice. For example, the synthesis of isopentyl diphosphate (IPDP), a primary precursor for lipid synthesis, can occur by one of two routes, the mevalonate pathway and the non-mevalonate pathway [35]. *B. subtilis* uses the non-mevalonate pathway for IPDP synthesis [36], [37]. The mevalonate pathway contains fewer reactions than the non-mevalonate pathway, and thus the parsimony-based gap filling approach incorrectly used the mevalonate pathway to restore IPDP production (**Figure 3**). However, all of the knocked out reactions in the non-mevalonate pathway had high estimated likelihoods. Hence, likelihood-based gap filling correctly chose this pathway to restore production of IPDP.

Given the extremely high magnitude of optimal penalties for parsimony-based gap filling and the more moderate magnitude of optimal penalties for likelihood-based gap filling, the optimal penalties for likelihood-based gap filling were selected as the defaults for the algorithm. These values were used for the remainder of the results in this manuscript. They are also the default parameters in the provided workflow script, though users are able to modify them at will.

### Likelihood-based gap filling produces more and higher-confidence annotations than post-processing

**Figure 4:**
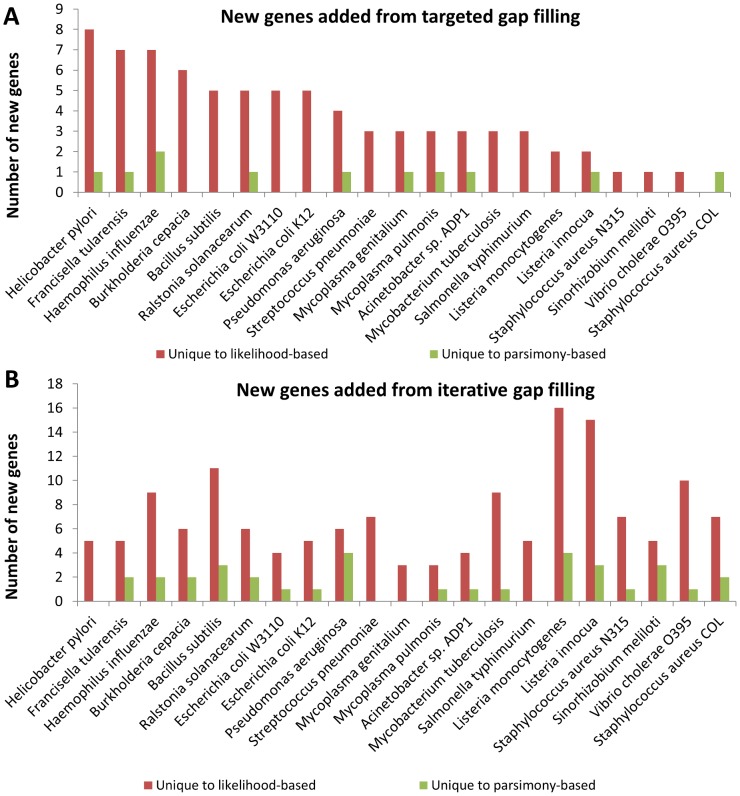
Genes added to the model using likelihood-based and parsimony-based gap filling. Likelihood-based gap filling produced more new gene annotations than post-processing gap filled reactions generated using the parsimony-based approach. The plot shows the number of uniquely-added genes by likelihood-based and parsimony-based gap filling approaches (genes in common with both approaches are omitted for clarity but tended to be more than those unique to either approach). A) Number of genes added after targeted gap filling to activate biomass production. B) Number of genes added after iterative gap filling.

**Figure 5:**
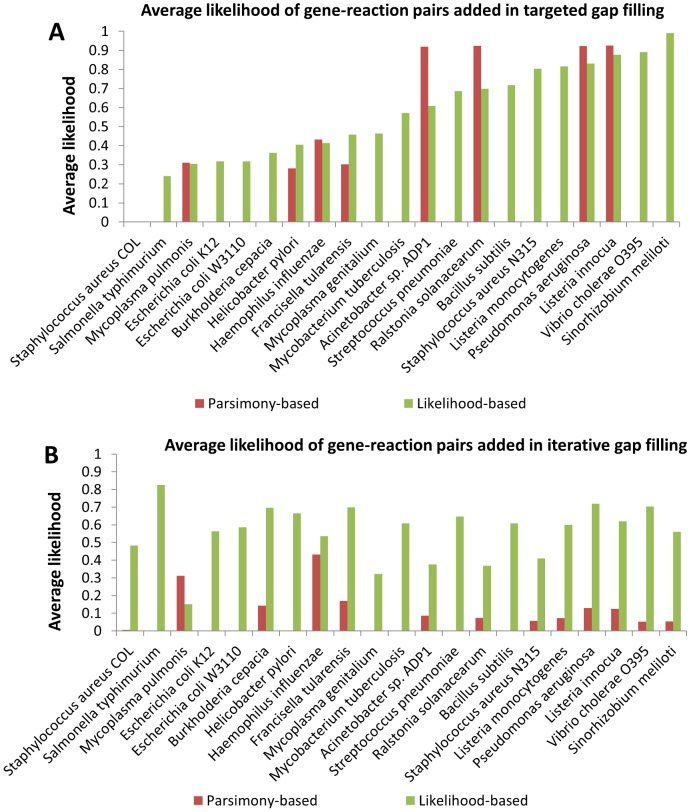
Likelihoods of gene-reaction associations added using likelihood-based and parsimony-based gap filling. The average likelihood of links between genes and reactions that were added using likelihood-based gap filling tended to be greater than the average likelihood of links resulting from post-processing the parsimony-based gap filling result. Note that it was not greater for all models (e.g., *Pseudomonas aeruginosa*) because the likelihood-based gap filling approach maximizes likelihood of reactions, not annotations, and as a result picks fewer reactions with 0 likelihood (no predicted gene associations). A) Targeted gap filling result. B) Iterative gap filling result.

One important step in curating gap filling solutions is identifying genes in the genome that could be responsible for catalyzing the gap filled reactions and assessing the quality of the genomic evidence behind these assignments [31]. Mapping between genes and reactions allows for a useful connection to genetic manipulations, drug targets, and experimental validation. We have compared the ability to identify genes with likelihood-based and parsimony-based gap filling by using the estimated Gene-Protein-Reaction relationships (GPR) from our likelihood computations to assign genes to reactions that are gap filled using each approach. We found that likelihood-based gap filling produced significantly more links between genes and reactions and more gene-associated reactions than post-processing of parsimony-based gap filling results (i.e. seeking for gene homology after reactions were gap filled). This was true for both the targeted gap filling and iterative gap filling approaches, though the effect was greater for iterative gap filling (*p*<10^−6^, Wilcoxon signed-rank test; **Figure 4**). By virtue of the optimization formulation, the average quality of annotation hypotheses generated from gap filling was also significantly greater for likelihood-based gap filling, as measured by computing the average likelihood of gene associations added using the likelihood-based approach vs. post-processing parsimony-based gap filling solutions (p<0.01, Wilcoxon signed-rank test; **Figure 5**). Therefore, the likelihood-based approach yields a larger quantity of better-supported candidate annotations for genes than simply searching for genes associated with parsimony-based gap filling results *post-hoc*.

### Likelihood-based and parsimony-based gap fill have similar consistency with experimental phenotypes

One commonly-used method to verify the integrity of genome-scale metabolic models is to compare their predictions with high-throughput phenotyping data, such as knockout lethality screens [13]. To test the impact of each of our workflows on the accuracy of model phenotype predictions, we applied our workflows to construct and fill gaps in genome-scale models for 22 organisms for which either Biolog or gene knockout lethality data was available. We then compared the predictions of these models to the phenotype data, without fitting to the data (**Table 1**). There were many differences in the pathways identified using likelihood-based gap filling compared to parsimony-based gap filling: between 5% and 30% of the reactions in a likelihood-based gap filling solution were not found in the parsimony-based solution, despite using the same parameters for each (see **Text S1**). However, the use of likelihoods did not significantly affect the phenotype predictions. For Biolog data, iterative gap filling increased the aggregate sensitivity by 11% compared to targeted gap fill, but decreased aggregate specificity (more false positives) by 10%–13%. The aggregate accuracy decreased by about 1% for iterative compared to targeted gap filling.

**Table 1:** Average phenotype consistency across all test organisms for models gap filled using the four evaluated algorithms.

	Biolog data		Essentiality data	
	Sensitivity	Specificity	Sensitivity	Specificity
Targeted parsimony-based	56%	69%	86%	67%
Targeted parsimony PP	56%	69%	84%	68%
Targeted likelihood-based	56%	69%	84%	67%
Iterative parsimony-based	66%	59%	86%	64%
Iterative parsimony PP	66%	59%	85%	64%
Iterative likelihood-based	67%	56%	85%	65%

**Figure 6:**
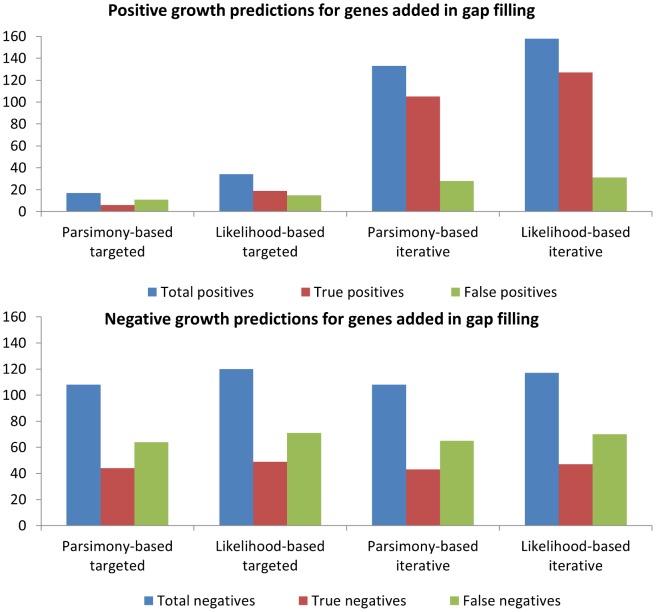
Knockout lethality accuracy for genes added in gap filling. Gene knockout simulations were performed for models gap filled with each of the four workflows to assess the consistency between lethality prediction and knockout lethality data for genes added in gap filling. Likelihood-based gap filling was able to produce the most candidate gene associations, with high specificity and low sensitivity in lethality predictions. The difference in accuracy between likelihood-based and parsimony-based gap filling was not statistically significant. A) Number of positive growth predictions, B) Number of negative growth predictions.

Iterative gap filling greatly increased the sensitivity (more correct positive growth conditions) and reduced the specificity (more incorrect positive growth conditions) of Biolog simulations. The use of likelihoods did not have a significant effect on the specificity or sensitivity of Biolog simulations. The overall model accuracy for essentiality data was similar for all four algorithms because genes added due to likelihood-based gap filling represented only at most about 7% of the genes in the model. See Figure 6 for the results of knockout simulations using only genes added to gap filling solutions. “PP” means post-processed to add genes to gap filled reactions.

Since gap filling only adds a small number of genes to the model compared to the number in the draft model (about a 6% increase for iterative gap filling), the sensitivity and specificity of knockout lethality predictions were very similar for all four workflows. The aggregate sensitivity was 84–86% for all four workflows while aggregate specificity was 64–68%. We also examined the lethality predictions specifically for genes added in gap filling (**Figure 6**). The negative predictive value was essentially identical for all four workflows at 40%. However, there was a notable improvement in the positive predictive value in the iterative gap filling workflows (80%) compared to targeted gap filling workflows (35% for parsimony-based and 55% for likelihood-based gap filling). Taken together, these results indicate that iterative gap filling mostly adds genes predicted to be nonlethal knockouts, and that most of these predictions are correct.

## Discussion

An important feature of the likelihood-based gap filling algorithm is that it can differentiate between genomes by assigning organism-specific likelihoods for each reaction in a network. As a direct result, the gap filling solutions resulting from this algorithm are also organism-specific. This direct link back to evidence in the genome directly enables the identification of pathways that are not parsimonious, but that are most consistent with genomic data. We have shown that the likelihood-based approach increases both the quality and the quantity of hypothesized gene associations from gap filling, especially when using the iterative approach to maximize the number of activated reactions subject to evidence constraints. Of course, when building a high-quality network model, gap filled pathways should be evaluated by experts to evaluate the evidence cited in the algorithm, to search for existing experimental evidence in favor of or refuting the suggested solutions, or to design new experiments to test the existence of the hypothesized functions in the modeled organism [13], [21]. The reported confidence metrics for annotations and for reactions will help curators target these curation efforts.

The likelihood-based gap filling methods described in this manuscript use genomic evidence-based metrics for the confidence that can be placed in annotations and reactions. The pathways that result from maximizing confidence have a greater genomic coverage and stronger evidence for inclusion of genes compared with the common procedure of post-processing parsimony-based solutions. Therefore, including likelihoods into the gap filling procedure directly improves on the state of the art in evaluating and selecting gap filling solutions.

Despite the significantly increased level of evidence for gap filling solutions resulting from likelihood-based gap filling, we did not observe a significant difference from existing approaches in the accuracy of knockout lethality or growth (Biolog) predictions. This result suggests that using phenotype data to filter gap-filling solutions may not result in a more accurate metabolic network (that is, one that better reflects biological evidence for the specific components included). Indeed, validation metrics such as consistency with knockout lethality predictions have a large number of ways in which they could be fit to become consistent with phenotype data, which can lead to decreases in observed accuracy when the model is tested on new data not available during its construction [30]. This tendency to overfit models makes the use of reaction confidence metrics essential when evaluating discrepancies between models and phenotype data.

The proposed integration of likelihoods into gap filling can serve as a tool for hypothesis generation in biology. An initial pool of potential annotations with associated likelihoods can be generated using many different methods such as protein co-localization or co-occurrence [38], [39] or from high-throughput ‘-omics’ datasets such as metabolomics. This initial pool can be quite broad (many alternative functions for each gene). The likelihood-based gap filling approach we have outlined is sufficiently general to incorporate likelihoods based on any type of evidence. The gap filling algorithm then selects from this broad pool of hypotheses for the new annotations that best explain a complex combination of biological observations, providing insights into enzyme promiscuity, adaptation, and evolution.

Finally, the KBase framework in which all of our workflows are implemented helps address the need for a unified framework for systems biology. In addition to gap filling, KBase includes implementations of many other modeling and reconstruction tools such as tools for the automatic generation of compartmentalized community models [40] and phenotype reconciliation tools such as the gap generation algorithm implemented as part of the ModelSEED framework [25]. We anticipate that the use of likelihoods to guide solutions of these algorithms would also lead to better-supported networks and improvements in the ability to assess solution quality independently of the test data itself, thus reducing the impact of overfitting. As systems biology expands to incorporate a greater number of high-throughput biological measures, the utility of computational frameworks for leveraging this vast knowledge *in toto* becomes increasingly important.

## Methods

We have developed four workflows in the DOE KnowledgeBase framework: parsimony-based gap filling, likelihood-based gap filling, iterative gap filling, and likelihood-based iterative gap filling (**Figure 1**). We have included as part of the Supporting Information detailed tutorials on the use of the KBase web-based interface (**Text S2**) or the web service API (**Text S3**) to perform each of these workflows.

Parsimony-based and likelihood-based gap filling attempt to activate single inactive reactions in a network by some combination of reversibility changes and addition of reactions from a universal reaction database. Parsimony-based gap filling assigns costs for adding reactions or changing their reversibility based on the source database for the reaction, the estimated standard Gibbs free energy of reaction, and whether it is a transport reaction (see **Text S1**). Likelihood-based gap filling modifies the cost of adding reactions (but not the cost of reversibility changes) by incorporating an estimated likelihood of existence of a metabolic reaction in an organism. Reaction likelihoods are computed based on an estimate of the level of evidence for a gene annotation (e.g. annotation likelihoods) and a mapping from annotations to reactions (via protein complexes).

Iterative gap filling is the iterative application of either parsimony-based or likelihood-based gap filling approaches to activate the maximum possible number of reactions in a metabolic network. The iterative gap filling workflows include a reaction sensitivity analysis to prune redundant or poorly supported reactions (“non-contributing” reactions).

The following sections describe the parts of these workflows in detail.

### Building a genome object

The first step of each workflow (**Figure 1**) is importing an annotated genome into a workspace in the KBase system. KBase workspaces provide a way for users to store, share, and manage data objects that they have uploaded or generated by running KBase analyses. The genome data is imported into the workspace as a Genome typed object, which is a standardized format compatible with all KBase tools that expect genomes as input. All results from subsequent analyses are also stored in a KBase workspace as typed objects (of different types), and the methods used to generate them are tracked to ensure data provenance.

### Building a draft model

The annotations for genes in the genome are used to generate a draft model using the ModelSEED algorithm as previously described [20]. A description is available in **Text S1**.

### Computation of annotation and reaction likelihoods

The computation of reaction likelihoods for likelihood-based gap filling is achieved by first estimating the likelihood of multiple annotations for each gene in the query organism based on sequence similarity, and then by using mappings from annotations to reactions found in the ModelSEED reaction database [20] to convert these likelihoods into reaction likelihoods.

### Calculating annotation likelihoods

Annotation likelihoods are computed in reference to a database of genes with high-confidence annotations. For this purpose, we compiled a list of the protein sequences for all proteins whose function was either literature-supported or called as part of at least one SEED subsystem [41]. Functional annotations in the SEED subsystems are manually curated using multiple sources of information such as sequence similarity, phylogeny and gene context, and therefore represent a high-confidence reference set.

To minimize the amount of redundancy in the list of target proteins, they were binned into organism taxonomic units (OTUs) with roughly 97% 16S rRNA similarity [42]. The final target database included at most one protein from each OTU for each functional role. When possible, the representative protein was chosen from the representative organism of the OTU, which tends to be a better-understood organism with higher-quality annotations such as *Escherichia coli* K-12. If the representative organism for an OTU did not have a protein with that role in a subsystem or with a literature backing, a representative protein was chosen at random from another member of the OTU.

The computation of annotation likelihood scores was designed based on the principle that genes with more similar sequences are more likely to share the same function, but recognizing that these relationships are far from perfectly predictive [43]. The computation thus attempts to quantify the uncertainty in relation to the available database of high-confidence annotations by accounting for both the similarity of the query gene to genes in the reference database and the distribution of annotations of the reference genes (Figure S3).

Annotation likelihoods were calculated by first running BLASTP [44], [45] with an E-value cutoff of 10^−5^ against all of the genes in the high-confidence gene annotation data set. A log-score for each (query, target) pair was computed as:
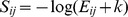

*E_ij_* is the E-value for the BLASTP hit between the protein products of genes *i* and *j* and *S_ij_* is the log-score between them. The parameter *k* = 10^−200^ was used to prevent the log E-value from being undefined due to a reported E-value of zero. After calculating log-scores for all (query, target) pairs, a likelihood score that each gene *i* ∈ *G_O_*, where *G_O_* is the set of genes in the organism, is also a member of the set *A_a_* of genes with annotation *a* was computed as follows:
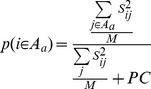

*A_a_* represents the set of genes with annotation *a*, 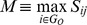 the maximum score of BLASTP hits from a gene in the query organism to a gene in the high-quality database, and *PC* = 40 is a pseudocount used to dilute the likelihoods of annotations for annotations with weak homology to the query. The sum in the numerator is over all BLASTP hits from gene *i* to genes with a particular annotation *a* and the sum in the denominator is over all BLASTP hits from gene *i*. Squaring the scores prevents large numbers of weak hits from dominating the computed likelihood.

The pseudocount was chosen to set the likelihood of a gene having moderately high homology (E = 1E-40) to a single protein in the database to 50% and is a typical parameter used in tools such as BLAST [45] to ensure that we offset for potentially incomplete or biased database representations of gene families. According to this formulation, *p*(*i* ∈ *A_a_*) will be high only if the protein product of gene *i* possesses strongly significant sequence similarity to reference proteins with *annotation a* and does not possess similarly strong similarity to proteins with other annotations. Therefore, the metric takes into account two different sources of annotation ambiguity: divergence of sequence and disparity of annotations for similar proteins in the target database.

### Calculating reaction likelihoods

Annotation likelihoods are useful for evaluating individual annotations, but must be converted into likelihoods for metabolic reactions in order to use them in the context of evaluating a metabolic network. Reaction likelihoods represent the confidence in the inclusion of a particular reaction in a metabolic network. The conversion from annotation likelihoods into reaction likelihoods takes into account the facts that annotations can imply multiple functional roles, a protein with a particular functional role could be part of multiple protein complexes, and multiple complexes could catalyze the same reaction. We used the ModelSEED reaction database [20] as the source of all of these links.

The first step in computation of reaction likelihoods is the conversion of gene annotations into functional roles, to account for the possibility that an annotation implies multiple protein functions. The likelihood that each gene *i* ∈ *G_O_* also belongs to the set of genes *R_r_* with functional role *r* was computed as the sum of the likelihood that gene *i* had each annotation that maps to role *r*.
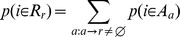
Here the mapping *a*→*r* from annotations to roles is defined by the ModelSEED database. This definition ensures that if a protein could be multi or single-functional, the final reaction likelihoods reflect each of those possibilities.

In the second step, the likelihood that *at least one* gene in *G_O_* had role *r* was computed as the maximum likelihood of the role across all genes in *G_O_*.
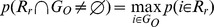
The genes most likely to fulfill role *r* (within 80% of the maximum) were retained and linked with an OR relationship to form a Boolean Gene-Function relationship.

In the third step, the ModelSEED reaction database was used to compute the likelihood of existence of protein complexes from the likelihood of existence of functional roles. A protein complex represents a set of protein functions that must all be present in order to build a multi-subunit enzyme (for example, an ATP binding subunit and a translocating subunit must both be present to build certain ABC transporters). Since all of the subunits must be present to perform the function, the likelihood of the existence of a complex *c* in the cell was computed as the minimum likelihood of the roles associated with it.
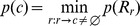
The mapping *r*→*c* from roles to complexes is provided by the ModelSEED reaction database. The sets of genes possessing each function in a complex were linked with an AND relationship to form a Boolean Gene-Protein relationship.

In the fourth step, reaction likelihoods were computed from protein complex likelihoods using complex-reaction links in the ModelSEED. Since multiple complexes can independently catalyze a reaction, the likelihood of the existence of a reaction *x* in the cell was computed as the maximum likelihood of the possible complexes that could catalyze it.
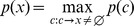
The complexes that could catalyze the same reaction were linked with an OR relationship to form a Boolean Gene-Protein-Reaction relationship (GPR) [46]. Only complexes with a likelihood within 80% of the maximum complex likelihood associated with a reaction were retained in the GPR for that reaction. The computed GPR was used in simulations of gene knockouts for gap filled reactions, and the reaction likelihoods were used as weights in the objective function for likelihood-based gap filling (see below)

### Targeted gap filling to complete media

In all workflows, targeted gap filling is performed first in order to activate the biomass equation and achieve growth on ‘complete’ media. Complete media consists of all compounds for which the organism has transport reactions in the draft reconstruction, and hence the solution to this gap filling problem represents reactions that would be necessary for growth on any more limited media for which the organism possesses transporters.

Targeted gap filling is performed using either the parsimony-based or the likelihood-based approach. The parsimony-based gap filling approach, used in the ModelSEED for auto-completing models [20], has been described previously [19], [25]. The parsimony-based approach minimizes the number of additions to a model. Penalties are added for use of less-confident biochemical databases, adding reactions with ambiguous compounds, adding of transport reactions, or making thermodynamically unfavorable reversibility changes. Higher penalties make it less favorable for the algorithm to make these types of modifications. Detailed descriptions of the formulation and penalties are available in **Text S1**.

The likelihood-based gap filling approach uses the same MILP formulation as parsimony-based gap filling. However, likelihood-based gap filling uses reaction likelihoods to re-weigh the objective coefficients. To do this, the likelihoods of reactions *p(x)* are first converted into costs *C(x)* by inverting them:
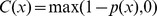
Then, modified gap filling objective coefficients *λ_gapfill_*_,*x*_ are computed as follows:
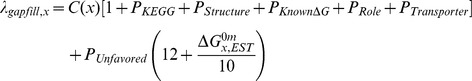
where *λ_gapfill_*_,*x*_ is the objective coefficient in the gap filling formulation for reaction x and the P-values represent the same penalties as used in the existing parsimony-based approach (see **Text S1**). In our modified formulation, higher-likelihood reactions are given lower costs (though the thermodynamic penalties for adding a reaction in the wrong direction are not changed) and are therefore favored in the optimization provided their benefit outweighs costs of other reactions in a pathway. The numeric parameters (12 and 10) in the equation make changing the reversibility of a reaction with low estimated Gibbs energy equivalent to adding (on average) one to two intracellular reactions in a favorable direction, while changing a reaction with reaction with an estimated Gibbs energy of 10 kCal/mol is equivalent to adding three average intracellular reactions in a favorable direction.

Due to the difficult nature of mixed-integer programs, obtaining a solution can take a long time for certain problems. Therefore, we have implemented a system in which a time limit is initially imposed on solution time and automatically increased if solving fails. An error is ultimately thrown if the solver fails to find a solution within one day or if no solution exists.

### Iterative gap filling

In iterative gap filling work flows, all reactions in the model that are associated with one or more genes are targeted to enable flux. Iterative gap filling is similar to the previously published gap find and gap fill algorithms [19], but operates on inactive reactions rather than dead-end or orphaned metabolites. This is accomplished by performing targeted gap filling on one reaction at a time until as many reactions as possible are functional. These targeted gap fillings can be performed using either the parsimony-based approach (parsimony-based iterative gap filling) or the likelihood-based approach (likelihood-based iterative gap filling).

The results of iterative gap filling depend on the order in which the targets are processed. In our studies, the order was selected based on the region of metabolism in which the reaction occurs. Central carbon reactions were gap filled first to ensure that core metabolism was functional. These were followed by reactions involved in biosynthesis of essential metabolites (amino acids, nucleotides, and cofactors), finally culminating in reactions involved in peripheral utilization and degradation pathways.

After ordering reactions according to this priority, flux variability analysis [47] was used to determine if each reaction had a non-zero maximum flux. If the maximum flux was zero, gap filling (likelihood or non-likelihood-based) was run to attempt to activate the reactions with pathways from the ModelSEED reaction database. If a gap filling solution was found, it was integrated into the model before moving onto the next reaction in the model. The final result was a set of pathways that activated a maximum number of reactions in the model.

### Reaction sensitivity analysis and deleting non-contributing reactions

Since iterative gap filling attempts to fill the maximum number of gaps in a model, solutions that fill different gaps in the model are often redundant or very poorly supported. To solve this problem, we have implemented a reaction sensitivity analysis that identifies for each gap filled reaction (including reversibility changes for existing reactions in a network): a) whether each gap filled reaction causes other reactions in the model to become inactive when it is removed and b) whether the gap filled reaction is predicted to be essential for growth. After performing reaction sensitivity analysis, any non-contributing reactions, which are non-essential gap filled reactions that do not activate any other reactions in the network, were removed from the model. For parsimony-based iterative gap filling, reaction sensitivity was performed on all reactions in the reverse order in which they were added, so that lower-priority gap filling solutions would be tested for removal first. For likelihood-based iterative gap filling, reaction sensitivity analysis was done in order from lowest to highest likelihood so that gap filled reactions that were unsupported by genetic evidence would be tested for removal first.

### Targeted gap filling to minimal media

In order to support simulations of Biolog data and achieve greater completeness of the metabolic network, the models gap filled on complete media are gap filled again to achieve non-zero biomass production on a minimal media. In this study Carbon-D-Glucose was used as the minimal media. This gap filling step can be performed using either the targeted or likelihood-based approach.

### Phenotype simulations

To perform phenotype simulations, high-throughput data is imported into the KBase workspaces and saved as PhenotypeSet objects in the workspace. These objects contain links from phenotype sets to specific media and genes in a genome, and represent the results of Biolog or knockout lethality experiments in a consistent format.

In this study, the ModelSEED algorithm [20] was used to build a draft model for each of 22 organisms for which either gene knockout lethality data (8 organisms), Biolog data (9 organisms), or both (5 organisms) was available [48]–[60]. All four gap filling workflows (targeted parsimony-based, targeted likelihood-based, iterative parsimony-based, and iterative likelihood-based) were independently applied to the draft model to build working models of each of these organisms. The gap filled models were verified to predict positive biomass production on Carbon-D-Glucose media using flux balance analysis [61] before performing further simulations.

To simulate gene knockout lethality phenotypes, the models growing on Carbon-D-Glucose media were first further gap filled (if necessary) to achieve nonzero biomass production on the media in which knockout experiments had been performed (this was only necessary for *Mycobacterium tuberculosis*). Subsequently, the knockouts were simulated by evaluating the Boolean GPR rules for each reaction and setting the maximum rate of each reaction whose GPR evaluated to FALSE to 0. Flux balance analysis was then used to maximize the biomass equation. The knockout was considered lethal if the predicted biomass production rate was less than 10^−9^ *hr* ^−1^. For parsimony-based gap filling knockout simulations were performed both before and after integrating predicted gene protein reaction associations for gap filled reactions into the model.

To simulate Biolog data, the models growing on Carbon-D-Glucose media were modified to possess transporters for every compound in every media in the Biolog array. After this modification, growth on each media was tested by setting exchange reactions for each compound not in the media to zero and using flux balance analysis to predict the biomass production rate. The model was considered non-growing if the predicted biomass production rate was less than 10^−9^ *hr* ^−1^.

### Workflow implementation details

All gap filling for this manuscript was performed outside of KBase using CPLEX under an academic license (IBM Corporation, version 12.5) [62]. Due to licensing restrictions, gap filling performed on KBase servers is done using SCIP 3.0.2 [63]. Phenotype simulations and sensitivity analysis were performed using GLPK version 4.43. The gap filling and likelihood computations are implemented in the KBase framework with web service APIs and a web interface (http://iris.kbase.us). Detailed descriptions of all steps in the workflow are available in **Text S2 and S3**.

Wilcoxon signed-rank tests were performed using the signrank function in MATLAB statistics toolbox version 7.1, using the ‘exact’ method and 2-tailed p-values.

## Supporting Information

Dataset S1 **Gap filling data summary.** Summary of gap filling data, including numbers and average likelihoods of added reactions and genes using the four workflows.
(XLS)

Dataset S2 **Parameter sensitivity analysis using iBsu1103.** Sensitivity of targeted gap filling results to changes in the unfavorable reversibility change and transporter penalties.
(XLS)

Dataset S3 **Analysis of genes unique to particular workflows.** Likelihoods and phenotype predictions for gene-reaction links specific to parsimony-based or to likelihood-based gap filling.
(XLSX)

Figure S1 **Likelihood of reactions added in gap filling.** Likelihoods of added reactions separated according to if they were only added in likelihood-based solutions, only added in parsimony-based solutions, or common to both solutions. Includes both iterative and targeted gap filling.
(PNG)

Figure S2 **Number of reactions unique to likelihood-based gap fill workflows.** Number of unique reactions to the likelihood-based workflow separated by organism.
(PNG)

Software S1 **Workflow client API.** Python code that may be readily installed and used to run the four complete workflows.
(ZIP)

Text S1 **Supplemental methods.** Descriptions of previously-published gap filling and model building methods implemented in the ModelSEED and elsewhere.
(DOCX)

Text S2 **Use of the KBase web interface.** Detailed documention of steps in the workflow and how to run each one using the KBase online command-line interface (http://iris.kbase.us).
(DOC)

Text S3 **Use of the KBase client API.** Documentation for use of the client API provided as Software S1.
(DOC)

Text S4 **Directions for use of the provided VM.** How to set up and run your own services to run the code.
(DOC)
