# Supplementary figures and images for: Likelihood-Based Gene Annotations for Gap Filling and Quality Assessment in Genome-Scale Metabolic Models
Source: PLoS Comput Biol. 2014 Oct 16;10(10):e1003882. doi: 10.1371/journal.pcbi.1003882 (PMC4199484; doi:10.1371/journal.pcbi.1003882)

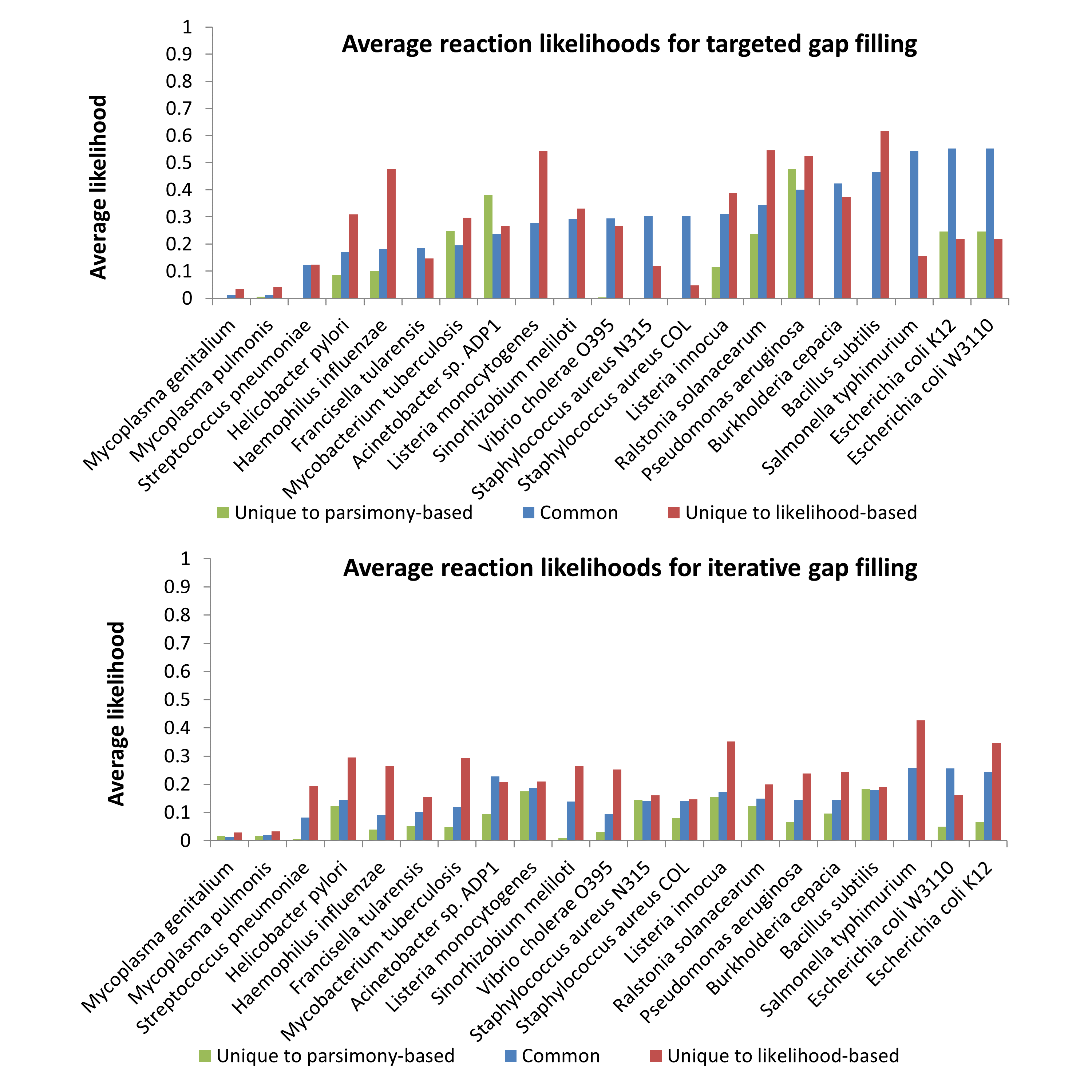

Supplement: Figure S1 — Likelihood of reactions added in gap filling. Likelihoods of added reactions separated according to if they were only added in likelihood-based solutions, only added in parsimony-based solutions, or common to both solutions. Includes both iterative and targeted gap filling. (PNG) [file pcbi.1003882.s004.png]

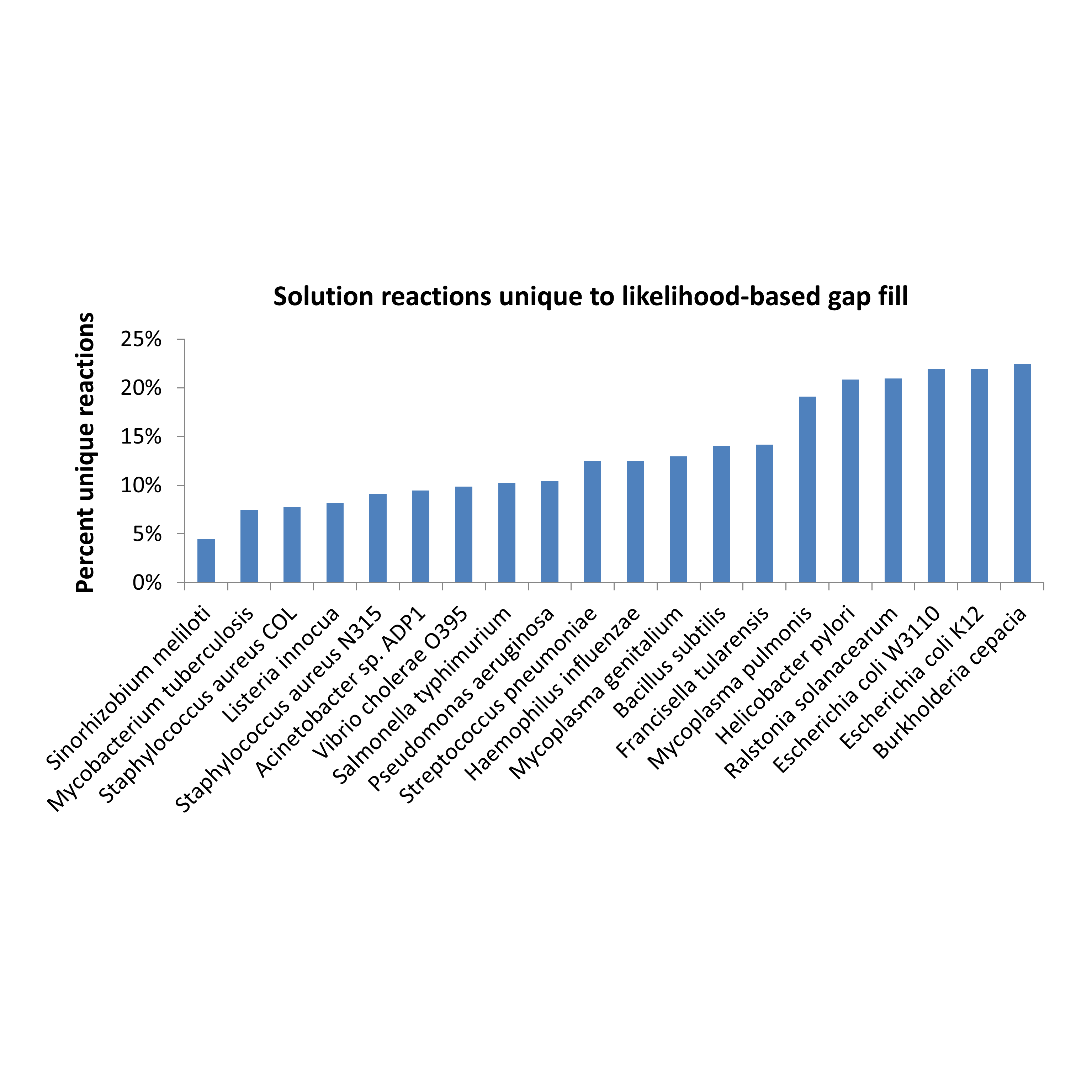

Supplement: Figure S2 — Number of reactions unique to likelihood-based gap fill workflows. Number of unique reactions to the likelihood-based workflow separated by organism. (PNG) [file pcbi.1003882.s005.png]
