# Supplementary material for: Likelihood-Based Gene Annotations for Gap Filling and Quality Assessment in Genome-Scale Metabolic Models
Source: PLoS Comput Biol. 2014 Oct 16;10(10):e1003882. doi: 10.1371/journal.pcbi.1003882 (PMC4199484; doi:10.1371/journal.pcbi.1003882)
Supplement: Text S1 — Supplemental methods. Descriptions of previously-published gap filling and model building methods implemented in the ModelSEED and elsewhere. (DOCX) [file pcbi.1003882.s007.docx]

**Text S1: Supplemental Methods**

**Building draft metabolic models**

Draft metabolic models were built using the procedure outlined in a previous manuscript [1]. Genomes were first annotated using vocabulary controlled in the SEED subsystems [2]. Then a biochemistry database maintained in the ModelSEED was used to translate these annotations into gene functions, protein complexes, and finally reactions predicted to be present in the reconstructed organism. The ModelSEED biochemistry database is based on KEGG and 13 previously-published, manually-curated genome-scale models (see manuscript [1] for details). Universal and spontaneous reactions such as diffusion of carbon dioxide were automatically added to the draft network. The reversibility of reactions in the draft networks were determined using thermodynamic predictions based on a group contribution method [3]. Thermodynamic estimates were performed on a 1mM basis at $25^o C$, 1 atm and a pH of 7.

**Parsimony-based Gap Filling**

The mixed-integer linear programming (MILP) formulation for parsimony-based gap filling has been published previously [4, 5]:

$$Minimize \sum\lambda_{gapfill,x}z_{x}$$

$$Subject to$$

$$\boldsymbol{N}\nu=0$$

$$0\leq\nu_{x}\leq v_{max,x}z_{x}$$

$$\nu_{target}\geq\epsilon$$

Here, ε=10^-3^, **N** is the stoichiometric matrix for all reactions in the biochemistry database from which gap filled reactions are drawn, ν is the reaction rate, v_max,x_ is the maximum reaction rate for reaction x, and z_x_  is 1 if the reaction x is added to the model and 0 otherwise. v_target_ is the reaction rate of a target reaction which is to be activated by the gap fill algorithm. The objective coefficient λ_gapfill,x_ is computed as [1]:

$$\lambda_{gapfill,x}=1+P_{KEGG}+P_{STRUCTURE}+P_{TRANSPORT}+P_{ROLE}+P_{Known \Delta G}+$$

$$P_{UNFAVORED}*(12+\frac{\Delta G_{x,EST}^{0m}}{10})$$

The first five P-values are penalties for adding reactions not in the KEGG database, unknown structures, transporters, missing roles, or for which the Gibbs free energy could not be estimated using a group contribution method. The final coefficient $P_{UNFAVORED}$ penalizes the addition of reactions in the predicted thermodynamically-unfavorable direction. $\Delta G_{x,EST}^{0m}$is the estimated Gibbs free energy of reaction by the group contribution method [3].

The values of the penalties used in this manuscript were as follows (though they can be adjusted by the user).

- P_KEGG­_: 0 for reactions in KEGG and 1 for other reactions,
- P_STRUCTURE_: 0 for reactions with only metabolites with known structure and 1 otherwise
- P_TRANSPORT_: 25, which works out to about 3-4 internal reaction changes on average.
- P_Known delta G_: 0 for reactions with estimated Gibbs energy and 1 otherwise
- P_UNFAVORED_: 0 if the reaction is in the thermodynamically favorable direction (or if it is predicted to be reversible) and 1 otherwise. This makes changing a reaction with an estimated Gibbs energy of 10 kCal/mol equivalent to adding (on average) three intracellular reactions in a favorable direction.

The same values of the parameters were used for parsimony-based and likelihood-based gap filling; thus the likelihood-based algorithm reduces to the parsimony-based algorithm in the limit of 0 likelihood for every reaction.

**References**

1. Henry CS, DeJongh M, Best AA, Frybarger PM, Linsay B, Stevens RL: **High-throughput generation, optimization and analysis of genome-scale metabolic models**. *Nat Biotechnol* 2010, **28**(9):977-982.

2. Overbeek R, Begley T, Butler RM, Choudhuri JV, Chuang HY, Cohoon M, de Crecy-Lagard V, Diaz N, Disz T, Edwards R *et al*: **The subsystems approach to genome annotation and its use in the project to annotate 1000 genomes**. *Nucleic Acids Res* 2005, **33**(17):5691-5702.

3. Jankowski MD, Henry CS, Broadbelt LJ, Hatzimanikatis V: **Group contribution method for thermodynamic analysis of complex metabolic networks**. *Biophys J* 2008, **95**(3):1487-1499.

4. Henry CS, Zinner JF, Cohoon MP, Stevens RL: **iBsu1103: a new genome-scale metabolic model of Bacillus subtilis based on SEED annotations**. *Genome Biol* 2009, **10**(6):R69.

5. Kumar VS, Dasika MS, Maranas CD: **Optimization based automated curation of metabolic reconstructions**. *BMC Bioinformatics* 2007, **8**:212.
