# Supplementary material for: Likelihood-Based Gene Annotations for Gap Filling and Quality Assessment in Genome-Scale Metabolic Models
Source: PLoS Comput Biol. 2014 Oct 16;10(10):e1003882. doi: 10.1371/journal.pcbi.1003882 (PMC4199484; doi:10.1371/journal.pcbi.1003882)
Supplement: Text S2 — Use of the KBase web interface. Detailed documention of steps in the workflow and how to run each one using the KBase online command-line interface (http://iris.kbase.us). (DOC) [file pcbi.1003882.s008.doc]

**Tutorial for building models and running likelihood-based gap filling in the DOE KnowledgeBase**

**Overview**

This tutorial describes how to build metabolic models using the four workflows that we have described in the manuscript. There are actually two ways to run this workflow:

1. Using the **Client API**, or
2. Using the **Web-based command line interface**

This tutorial focuses on the second one. The web-based command line interface is available at:

[http://iris.kbase.us](http://iris.kbase.us/)

Users log in (signing up is easy and free), create a workspace, and run the commands sequentially in the provided window. The API is described in a separate tutorial.

This tutorial is divided into three parts: A quick command reference (commands are outlined with minimal explanation), a brief overview of the KBase infrastructure necessary to run the commands, and detailed information about each step we ran in the manuscript to come up with the reported results.

Depending on the type of gap filling you wish to run there are a total of up to 13 steps. Below are detailed workflows for reproducing our results from the manuscript (or running your own) with parsimony-based gap filling, iterative gap filling, likelihood-based gap filling, and likelihood-based iterative gap filling. Look up the appropriate section below for a detailed description of the commands for each step. Skip over anything in gray for a particular analysis.


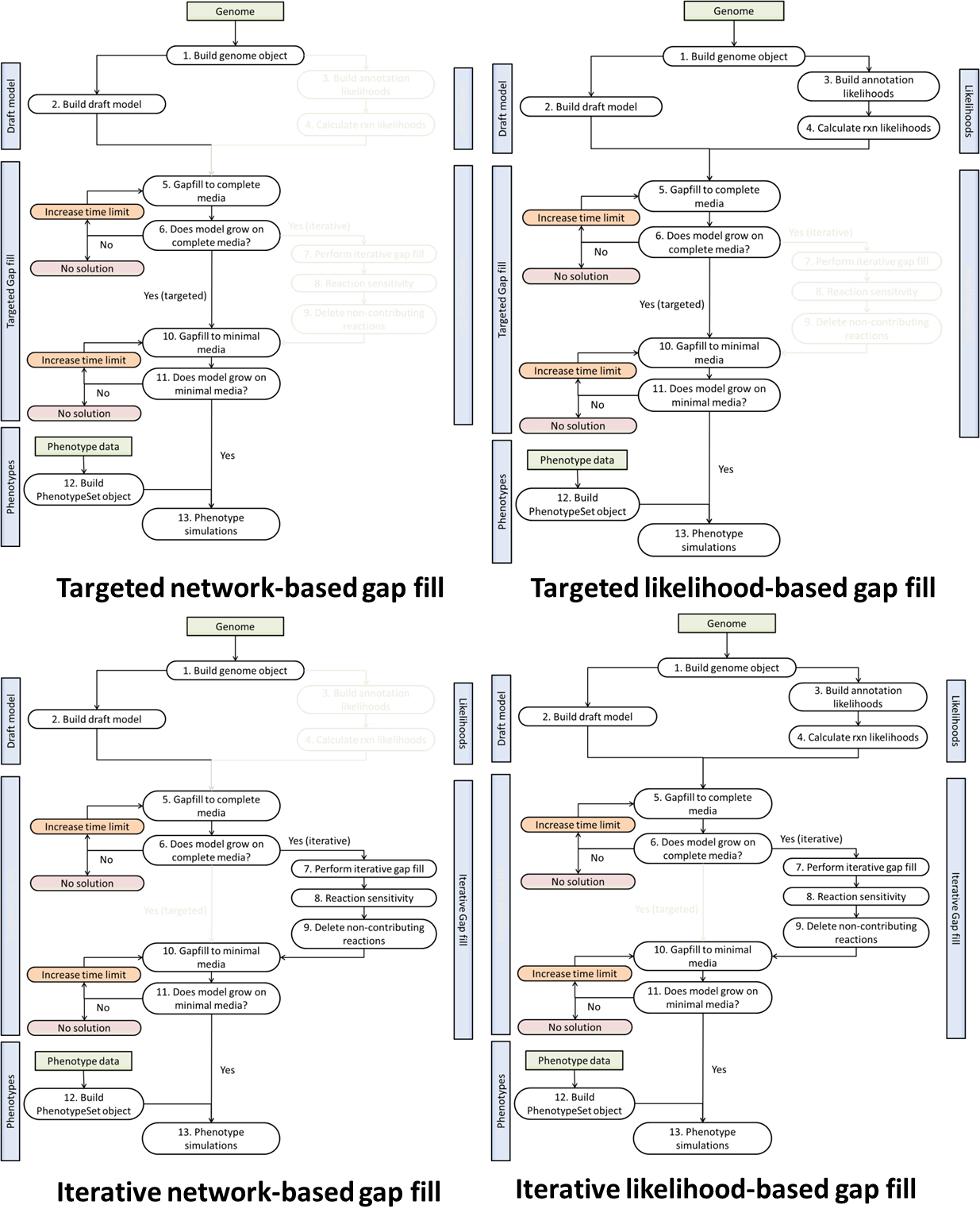


**Quick command reference**

The following is a list of all the commands (in order) that you can run in IRIS to perform a complete analysis from loading a genome from the SEED to performing a likelihood-based gap filling and analyzing phenotype data (in the same manner as was done in the manuscript). The numbers here correspond to the numbers in the workflows above. Anything denoted as $STUFF should be replaced with the actual name of the desired inputs and outputs.

In order to run any of these commands you will need to first need a Globus Online account. If you dont have one, create one here:

<https://gologin.kbase.us/SignUp>

Go to IRIS ([http://iris.kbase.us](http://iris.kbase.us/)) and log in. You will then need a workspace in which to store your data. If you don't have one, create one using:

$ ws-createws $WORKSPACE_NAME

Then switch to that workspace using:

$ ws-workspace $WORKSPACE_NAME

**1**. **Build a genome object (this example imports the genome from the PubSEED, other options are availbale)**

$ fba-loadgenome --seed $SEED_ID

This will create a Genome object called $SEED_ID in your workspace (this is hereafter referred to as $GENOME_OBJECT_NAME since other sources are possible)

**2. Build draft model**

$ fba-buildfbamodel $GENOME_OBJECT_NAME --model $DRAFT_MODEL_NAME

This will create a Model object called $DRAFT_MODEL_NAME in your workspace.

**3. Build annotation likelihoods**

$ pa-annotate $GENOME_OBJECT_NAME $PROBANNO_OBJECT_NAME

This will create a ProbAnno object called $PROBANNO_OBJECT_NAME in your workspace. This is a long-running job (takes about 4-5 hours on average). You can check the status of your job using

$ pa-checkjob --job 52efbcb7e4b0ef8357332113

Job '52efbcb7e4b0ef8357332113' (pa-annotate for genome 171101.1.genome to probanno 171101.1.probanno for user mmundy) has status 'running blast' and is working on task 3 of 5.  Check again later.

**4. Calculate reaction likelihoods**

$ pa-calculate $PROBANNO_OBJECT_NAME $RXNPROBS_OBJECT_NAME

This will create a RxnProbs object called $RXNPROBS_OBJECT_NAME in your workspace.

**5. Fill gaps on complete media**. How you do this depends on the type of gap filling you want to do. This is a long-running job (takes from 1 hour to 1 day depending on the number of gaps in the original network).

**Parsimony-based gap fill**:

$ fba-gapfill $DRAFT_MODEL_NAME --modelout $GAPFILLED_MODEL_NAME \

--transpen 25 --singletranspen 25 --biomasstranspen 25 --directionpen 12 \

--intsol

**Likelihood-based gap fill:**

$ fba-gapfill $DRAFT_MODEL_NAME --modelout $GAPFILLED_MODEL_NAME \

--transpen 25 --singletranspen 25 --biomasstranspen 25 --directionpen 12 \

--intsol --probrxn $RXNPROBS_OBJECT_NAME

**6. Check growth on complete media**

$ fba-runfba $GAPFILLED_MODEL_NAME --fbaid $FBA_OBJECT_NAME.

Should give you non-zero objective value. The results are stored in an FBA object named $FBA_OBJECT_NAME for future reference.

**7. If you are doing iterative gap filling**, run the following after doing all the above for targeted gap filling (this is a long-running job, it takes 2-3 days on average to run):

**Iterative gap fill:**

$ fba-gapfill $GAPFILLED_MODEL_NAME --modelout $ITER_GAPFILLED_MODEL_NAME \

--transpen 25 --singletranspen 25 --biomasstranspen 25 --directionpen 12 \

--intsol --iterativegf

**Likelihood-based iterative gap fill:**

$ fba-gapfill $GAPFILLED_MODEL_NAME --modelout $ITER_GAPFILLED_MODEL_NAME \

--transpen 25 --singletranspen 25 --biomasstranspen 25 --directionpen 12 \

--intsol --iterativegf --probrxn $RXNPROBS_OBJECT_NAME

Note that this job can take up to a couple days for some problems. The final result will be a new Model object $ITER_GAPFILLED_MODEL_NAME in the workspace.

**8. Reaction sensitivity analysis (iterative gap fill only)**

To run a reaction sensitivity analysis you need a GapFill solution ID. Run this:

$ fba-getmodels --pretty $gapfilled_model_name $WORKSPACE_ID \

> $gapfilled_model_filename

Then search for "integrated_gapfillings" in the output file .

"integrated_gapfillings" : [

[

"kb|g.166872.fbamdl1.gf.3",

"652/14/1",

"Complete",

"262/34/1",

0,

[]

]

],

You want the first element of the array ( kb|g.166872.fbamdl1.gf.3 in this example). Add "gfsol.0" to that string to get the GapFill solution ID (note - the 0 means you want to integrate solution number 0, i.e. the first solution):

kb|g.166872.fbamdl1.gf.3.gfsol.0

For **iterative gap fill**:

$ fba-reactionsensitivity $ITER_GAPFILLED_MODEL_NAME \

--rxnsensid $RXN_SENSITIVITY_NAME \

--gapfill $GAPFILL_SOLUTION_ID --deleterxns

For **likelihood-based iterative gap fill**:

$ fba-reactionsensitivity $ITER_GAPFILLED_MODEL_NAME \

--rxnsensid $RXN_SENSITIVITY_NAME \

--gapfill $GAPFILL_SOLUTION_ID --deleterxns --rxnprobs $RXNPROBS_OBJECT_NAME

The result will be a RxnSensitivity object $RXN_SENSITIVITY_NAME in your workspace.

**9. Delete non-contributing reactions**

$ fba-delete_noncontributing_reactions $RXN_SENSITIVITY_NAME \

--newmodel $FILTERED_MODEL_NAME

It will make new Model object $FILTERED_MODEL_NAME in your workspace with flagged reactions deleted.

**10. Gapfill to minimal media**

We used Carbon-D-Glucose but other minimal media will work as well. For **parsimony-based** (targeted or iterative) gap fill, use the following to fill gaps to Carbon-D-Glucose (do not use iterative gapfill again on minimal media even if you did it on complete media):

$ fba-gapfill $INPUT_MODEL_NAME --modelout $MINIMAL_GAPFILL_MODEL_NAME \

--transpen 25 --singletranspen 25 --biomasstranspen 25 --directionpen 12 \

--intsol --media Carbon-D-Glucose --mediaws KBaseMedia

For **likelihood-based** or **likelihood-based iterative** use the following:

$ fba-gapfill $INPUT_MODEL_NAME --modelout $MINIMAL_GAPFILL_MODEL_NAME \

--transpen 25 --singletranspen 25 --biomasstranspen 25 --directionpen 12 \

--intsol --probrxn $RXNPROBS_OBJECT_NAME --media Carbon-D-Glucose \

--mediaws KbaseMedia

Depending on whether you have are doing targeted or iterative gap fill workflows, $INPUT_MODEL_NAME should be $GAPFILLED_MODEL_NAME or $FILTERED_MODEL_NAME, respectively.

**11. Check for growth on minimal media**

$ fba-runfba $MINIMAL_GAPFILL_MODEL_NAME --fbaid $MINIMAL_FBA_OBJECT_NAME \

--media Carbon-D-Glucose --mediaws KBaseMedia

Like before, you should get a non-zero objective. If it fails try rerunning gap filling with higher time limits.

**12. Import phenotype data**

Note - we already loaded the phenotype data cited in the manuscript in the KBasePhenotypeDatasets directory so if you want to use that data you can skip this step.

Compile your phenotype data into a tab-delimted table (see detailed description below). Then run:

$ fba-importpheno $GENOME_ID $YOUR_PHENOTYPE_FILE \

--phenoid $PHENOTYPE_SET_ID

The function will create a PhenotypeSet object $PHENOTYPE_SET_ID in your workspace.

**13. Do phenotype simulations**

**For biolog data** make sure you specify to add transporters for growth conditions. The input model $MODEL_NAME should be the model that has been gap filled to grow on minimal media.

$ fba-simpheno $MINIMAL_GAPFILL_MODEL_NAME $PHENOTYPE_SET_ID \

--phenows KBasePhenotypeDatasets \

--phenosimid $OUTPUT_SIMULTATIONS --alltransporters

**For knockout data** just make sure the model grows on the media in which knockouts were done (if not, do a gap filling to that media - step 9-10, but replace Carbon-D-Glucose with your media ). Then run:

$ fba-simpheno $MINIMAL_GAPFILL_MODEL_NAME $PHENOTYPE_SET_ID \

--phenows KBasePhenotypeDatasets \

--phenosimid $OUTPUT_SIMULATIONS

These commands will create a PhenotypeSimulationSet object called $OUTPUT_SIMULATIONS in your workspace, storing all the simulation results.

----------------------------------------------------------------------------------------------------------------------------------

**A very brief introduction to the KBase**

The DOE KnowledgeBase (KBase) is, to me, three things:

a. A database with consistent identifiers, cross-linked to show connections between genomes, genes, functions, and (perhaps most importantly) biochemistry. We won’t talk about this much in the demo but it does make it much easier to import external data and have it generate the right links...

b. A set of tools (particularly, modeling tools) that can be used to analyze that data, along with a set of consistent APIs that can be used to develop your own tools and a web front-end that can run them from anywhere

c. A provider of computational resources such as processing and storage

In the KBase your data will be saved on KBase machines (which are backed up regularly) and processed on their servers. All of the below commands are run in a web environment hosted by the KBase team.

**A. Sign up for a KBase account.**

To use the KBase you will need to sign up for an account through Globus Online. Do so through their website:

<https://gologin.kbase.us/SignUp>

**B. IRIS**

IRIS is a web-based command line tool, located here:

[http://iris.kbase.us](http://iris.kbase.us/)

I’ll show you some commands you can use to build models in IRIS - you just type them in and they run on some computer in KBase-land. No installation necessary.

To begin using IRIS, you need to log in using your Globus Online account.

After you log in, you can upload your data, run commands with it, and export the data back to your computer. If you log in on another computer, your data will still be there. A nice tutorial on the interface for IRIS is available here so I won't repeat the words from it. I recommend you read it to become familiar with how to upload and download files, run commands etc.

<http://kbase.science.energy.gov/developer-zone/tutorials/iris/introduction-to-the-kbase-iris-interface/>

**C. Workspaces and objects (KBase data storage)**

After you upload your data you will need to run a script to convert it into a KBase object. For example, there is a command to take a FASTA file and turn it into a **genome object**, to take a SBML file and turn it into a **model object**, etc. There are also interfaces to automatically download data from various databases (such as the SEED) and save them as KBase objects.

All KBase objects are given a specific type and saved in a **workspace** (think of it like a folder) on the KBase computers. Most KBase commands take an object of one type and convert them into another object of the same type or an object of a different type (for example, there is a command to convert a genome object into a model object).

In order to move forward you will need to create a workspace to store your files. Do that with the **ws-createws** command. Type the following into IRIS to create a workspace, replacing $WORKSPACE_NAME with the name of the workspace you want to create (which by default no one but you can read. You can always change permissions later):

$ ws-createws $WORKSPACE_NAME

Then change to that workspace using **ws-workspace**

$ ws-workspace $WORKSPACE_NAME

You are now in your workspace $WORKSPACE_NAME. You can list the objects in that workspace at any time by typing **ws-listobj**

$ ws-listobj

To list objects in a specific workspace (not the one you're currently in) use -w. For example use this command to list all the media in the KBaseMedia workspace.

$ ws-listobj --workspace KBaseMedia

The functions used in this workflow can take both an ID and a workspace for any of the objects that they require as inputs. They usually will use your current workspace by default if you don't specify it; this is omitted in the example commands below, in which we assume you are currently in the workspace that you wish to save objects into.

**D. KBase services**

A **service** is basically a collection of related commands (often operating on just a few different types of objects). There are lots of services in the KBase for genome annotation, clustering and orthology analysis, analysis of transcription data, etc. I haven’t used most of them myself. To do likelihood-based gap filling, you will need to use commands in three of them:

- The **workspace service**, which is used to save, move, and retrieve data from workspaces (think of them as personal folders on KBase machines that store objects with specific formats). You'll need to use this with practically every other service.

- The **modeling service,** which has functions to import and annotate genomes, import existing models or generate new ones from an annotated genome, run gap filling, compare models, etc. It uses the ModelSEED as a back-end

- The **probabilistic annotation service**, which calculates annotation and reaction likelihoods for use in gap filling.

In IRIS you can see the list of services in the tab on the left. I’ve boxed the modeling and workspace services in the figure below for your reference.


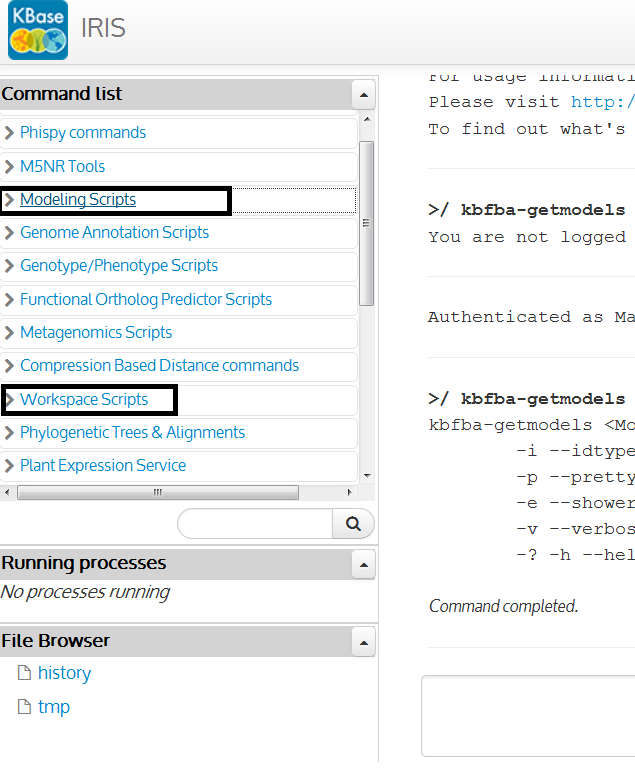


---------------------------------------------------------------------------------------------------------

**Detailed workflow tutorial**

The following is a detailed description of the relevant commands to run our workflow in IRIS. Anytime you see $STUFF , replace it with the actual name of your input and outputs.

**1. Build genome object**

In order to work with your genome in the KBase you will need to import it into a workspace. Genomes, like everything else in workspaces, are saved as **typed objects** which have formats that all of the KBase functions understand and store all necessary information.

Fortunately, doing this conversion is rather simple, especially for genomes in the SEED, the KBase central store, or RAST. Note that for model building to work properly you will need to be using the same annotation conventions as are used in the SEED\RAST\KBase central store so it is highly recommended that you take your genomes from one of those sources. For example, pull a genome from the SEED by its SEED ID using:

$ fba-loadgenome --seed $SEED_ID

This will create a Genome object called $SEED_ID in your workspace (this is hereafter referred to as $GENOME_OBJECT_NAME since other sources are possible). Take a look at the other options for fba-loadgenome if your source is different.

**2. Build draft model**

After you load your genome into a workspace, you can use the ModelSEED algorithm (**Ref:** see Henry *et al.* 2010) to build a draft model using:

$ fba-buildfbamodel $GENOME_OBJECT_NAME --model $DRAFT_MODEL_NAME

This will create a Model object called $DRAFT_MODEL_NAME in your workspace based on the annotations in the genome object.

The draft model is *not yet gap filled* so it will not grow due to missing or incorrect annotations. However, a biomass equation will automatically be created and after gap filling is done (see later steps) it should be able to grow provided the solving did not fail.

**3. Build annotation likelihoods**

The **pa-annotate** command (in the Probabilistic Annotation service on IRIS) is responsible for computing the likelihoods of annotations for each gene in a genome as described in the manuscript. It takes a genome object as an input and produces a ProbAnno object:

$ pa-annotate $GENOME_OBJECT_NAME $PROBANNO_OBJECT_NAME

Running this command will result in queueing a probanno job on the KBase servers. The command takes about 4 hours to run on average, and therefore the job is placed in a queue and runs when the queue is cleared. You can always check on your job using ws-checkjob:

$ pa-checkjob $JOB_ID

When the job is complete, a ProbAnno object called $PROBANNO_OBJECT_NAME will be created in your workspace. This object will contain multiple possible annotations for each gene, each attached to a likelihood.

**4. Building reaction likelihoods**

The **pa-calculate** function takes a ProbAnno object as an input and calculates the likelihood of each reaction based on the procedure outlined in the manuscript. It is run as follows:

$ pa-calculate $PROBANNO_OBJECT_NAME $RXNPROBS_OBJECT_NAME

The result is a RxnProbs object called $RXNPROBS_OBJECT_NAME, which is saved in your workspace (unlike pa-annotate, pa-calculate runs quickly). The RxnProbs object stores computed reaction likelihoods along with the predicted gene-protein-reaction (GPR) relationships for each reaction and some data on the complexes used to build them.

You can use either a RxnProbs object or a ProbAnno object as an input to gap filling. These are the inputs to likelihood-based gap filling as described in the manuscript. However, we recommend using the RxnProbs object so that you can use it as input to other functions to help interpret gap filling results.

**5. Running Gap filling to Complete Media**

Draft models build using **fba-buildfbamodel** will not be able to simulate growth in general, due to the existence of annotation gaps. It is necessary to fill gaps in the model to achieve growth. Gap filling is done using the **fba-gapfill** command, which has many options (some of which were not used in this manuscript). The total commands used were as follows, which are described in detail below

**Parsimony-based gap fill**:

$ fba-gapfill $DRAFT_MODEL_NAME --modelout $GAPFILLED_MODEL_NAME \

--transpen 25 --singletranspen 25 --biomasstranspen 25 --directionpen 12 \

--intsol

**Likelihood-based gap fill:**

$ fba-gapfill $DRAFT_MODEL_NAME --modelout $GAPFILLED_MODEL_NAME \

--transpen 25 --singletranspen 25 --biomasstranspen 25 --directionpen 12 \

--intsol --probrxn $RXNPROBS_OBJECT_NAME

The following options to **fba-gapfill**  were *always* used in simulations done in this manuscript:

--transpen 25 --singletranspen 25 --biomasstranspen 25 --directionpen 12 \

--intsol

**--transpen, --singletranspen, --biomasstranspen**, and **--directionpen** are penalties for adding transporters for various things instead of intracellular reactions (I like to set these to be quite high but you can feel free to play with them) and the base penalty for changing reversibility if a reaction is not thermodynamically favorable in the reverse direction.

**--intsol** means "integrate solution"; gap fill will NOT automatically integrate the solution into your model unless you select this option. If you want to look at the solution and decide whether you agree before adding the reactions to your model, you can do so by omitting this option. We do not talk in this tutorial about how to integrate solutions after-the-fact but you can look at the fba-integratesolution command for some details.

When you run **fba-gapfill,** it will do some pre-processing and then will queue the job and give you an ID. Gapfill takes quite a while to run so go do something else for a day, come back and it should be done (if you are doing iterative gapfill, give it two or three days). You can always check the status of your job by using **ws-checkjob**.

If the solver fails and gives you a nonsense solution (e.g. 3000 reactions), try increasing the time per solution and total time using **--timepersol** and **--timelimit** (the default time limit for solving is 3600 seconds per solution).

**Likelihood-based gapfilling**

To do likelihood-based gapfilling as described in the manuscript, you need to either provide a **probabilistic annotation** object or a **probabilistic reaction** object (which contains the reaction likelihoods calculated from the probabilistic annotation object), which are calculated as described in **steps 3-4** of this tutorial.

The ProbAnno object can be incorporated directly into gapfilling (without calculating reaction likelihoods) with the **--probanno** flag to **fba-gapfill.**

However, we recommend using the **pa-calculate** function to compute them yourself and get a RxnProbs object so that you can take a look at the reaction likelihoods that are generated. The RxnProbs object can be used as input to gapfilling by providing the **--probrxn** flag to **fba-gapfill**.

**6. Checking growth on complete media**

You can run FBA within the KBase tool (along with many other simple simulations such as FVA, doing simulation to try to figure out what biomass components can't be produced in a model, etc…) using the **fba-runfba** command. I recommend doing this to make sure the gapfilling was successful in producing a growing model. The **fba-runfba** command will create an FBA object which stores the FBA results including the fluxes of every reaction (and if you ask for it, FVA results, results of running knockouts, etc. - I won't cover all of that stuff but take a look at the help text for an idea of what it can do).

I suggest saving the FBA object to a place where you know where to find it \ how it was run using the

**--fba** flag:

$ fba-runfba --fba $FBA_OBJECT_NAME $GAPFILLED_MODEL_NAME

The default is to run FBA on complete media so this is all you need to do to test if growth is possible. If the growth rate is 0, the gapfill failed. Depending on the reasons for failure you might be able to get a good solution by re-running gapfill with a higher time limit. Try increasing the time per solution and total time using **--timepersol** and **--timelimit** (the default time limit for solving is 3600 seconds per solution).

**7. Iterative gapfilling**

Iterative gapfilling, as described in the manuscript, is the gapfilling of ALL of the dead-ends in a model, not just those that have to be filled to achieve growth. It is called iterative because it fills them one a time according to a pre-defined priority until they are all filled (or as many as can be filled given what is in the database).

Here are some recommendations on how to use it for maximum effectiveness.

- You only need to ask for one solution (do not use **--**numsol. It will be ignored anyway)
- You should only do iterative gapfilling on **complete media** (which is the default - don't use the -m/--media or --mediaws flags).
- You should only do iterative gap fill **after** doing a normal gap filling (and integrating the solution and checking to make sure the model achieves growth on complete media). This greatly reduces the computation time necessary because gap filling to biomass makes many other gap fills unnecessary.
- Be aware that iterative gapfilling takes a long time because you're filling so many gaps. This is why we only use it when gapfilling to complete media and not when gapfilling to minimal media (**step 10**)
- Use **--intsol** - since you're only getting one solution you might as well just integrate it automatically. In fact, for iterative gapfill this is set whether you set it or not, but set it anyway so you don't forget.

To do an iterative gapfilling use the **--iterativegf** flag in the **fba-gapfill** function.

**Likelihood-based iterative gap filling**

Likelihood-based iterative gap filling can be done by providing both **--probrxn** (or --probanno) and

**--iterativegf** to the **fba-gapfill** function. Otherwise, use the same guidelines as for parsimony-based iterative gap filling.

The overall commands we used were as follows:

**Iterative gap fill:**

$ fba-gapfill $GAPFILLED_MODEL_NAME --modelout $ITER_GAPFILLED_MODEL_NAME \

--transpen 25 --singletranspen 25 --biomasstranspen 25 --directionpen 12 \

--intsol --iterativegf

**Likelihood-based iterative gap fill:**

$ fba-gapfill $GAPFILLED_MODEL_NAME --modelout $ITER_GAPFILLED_MODEL_NAME \

--transpen 25 --singletranspen 25 --biomasstranspen 25 --directionpen 12 \

--intsol --iterativegf --probrxn $RXNPROBS_OBJECT_NAME

**8. Reaction sensitivity analysis**

Reaction sensitivity analysis as described in the manuscript is a way to prune unneeded gap filling solutions by testing the effects of removing each one on the activity of other reactions in the network and on the ability of the cell to predict nonzero biomass production rates. If deleting a gap filled reaction is nonlethal and does not inactivate any reactions in the model, it is flagged as unnecessary. Reaction sensitivity analysis is done using the **fba-reactionsensitivity** function in IRIS. This function can be run in two different ways (only one of which is covered above, because it's the one we used when doing our simulations):

- The user can specify (in the order that he or she wants to test them) a list of reactions in the model for which the sensitivity should be tested. To do this use the **--rxnstotest** flag. For example, if rxn00001 and rxn00002 are in your model $MODEL_ID, you can test the effects of deleting them by running this command:

$ fba-reactionsensitivity $ITER_GAPFILLED_MODEL_NAME --rxnstotest "rxn00001;rxn00002"

You can also specify a direction (e.g. "+rxn00001") to test.

- The user can specify a **GapFill solution ID**. If the user specifies this, then the following happens:
  - A list is generated of all of the reaction changes (with the direction) added by that specific gap filling run.
  - For **parsimony-based gap fill** (no RxnProbs object specified), the order of reactions to test is the *reverse* of the order in which they were added by the algorithm, with the idea that later gapfills are to lower-priority parts of the network so we want to try to remove them first.
  - For **likelihood-based gap fill**, specify a RxnProbs object with **--rxnprobs** . A further stable sort will be done based on the reaction likelihoods and the lowest-likelihood reactions will be tested for removal first. Ties are broken by using the same ordering as for non-likelihood-based gap filling.

The process of getting a GapFill solution ID is as follows. Given a model that has an integrated iterative gapfill (using --intsol), run:

$ fba-getmodels –pretty $gapfilled_model_name $WORKSPACE_ID \

> $gapfilled_model_filename

Then search for "integrated_gapfillings" in the output file .

"integrated_gapfillings" : [

[

"kb|g.166872.fbamdl1.gf.3",

"652/14/1",

"Complete",

"262/34/1",

0,

[]

]

],

You want the first element of the array ( kb|g.166872.fbamdl1.gf.3 in this example). Add "gfsol.0" to that string to get the GapFill solution ID (note - the 0 means you want to integrate solution number 0, i.e. the first solution):

kb|g.166872.fbamdl1.gf.3.gfsol.0

**Optionally**, the reaction sensitivity analysis will delete each reaction that is unnecessary before proceeding to the next one (in this case, the reactions will be flagged for deletion in the Reaction Sensitivity object and the sensitivity results of every reaction after it will depend on the fact that that reaction was deleted). To get this behavior specify **--deleterxns** on the command line. We used this flag in the manuscript workflow.

Putting all of this together, the final commands we used as part of the manuscript workflow were:

**Parsimony-based gap fill**

$ fba-reactionsensitivity $ITER_GAPFILLED_MODEL_NAME --rxnsensid $RXN_SENSITIVITY_NAME

--gapfill $GAPFILL_SOLUTION_ID --deleterxns

For **likelihood-based gap fill**:

$ fba-reactionsensitivity $ITER_GAPFILLED_MODEL_NAME --rxnsensid $RXN_SENSITIVITY_NAME

--gapfill $GAPFILL_SOLUTION_ID --deleterxns --rxnprobs $RXNPROBS_OBJECT_NAME

**9. Deleting non-contributing reactions**

After you run a reaction sensitivity analysis with --deleterxns, you can run **fba-delete_noncontributing_reactions** to actually delete the unnecessary reactions from the model. The RxnSensitivity object is automatically linked to a specific model so you will not need to specify the input model in this function. However, you can (and probably should) specify a different ID to use for the model with reactions deleted. Do so with the -**-newmodel** flag.

$ fba-delete_noncontributing_reactions $RXN_SENSITIVITY_NAME \

--newmodel $FILTERED_MODEL_NAME

**10. Gap filling to minimal media**

We used the same commands as outlined in **step 5** (gap filling to complete media) except for two things:

1. By default, the gap fill algorithm only tries to achieve growth on "complete" media. You can specify other media using **-m** - you will also probably have to specify a media workspace. We recommend (and have implemented in this workflow) running gap filling on complete media first before trying to achieve growth on any specific media. Doing so greatly simplifies the gap filling problem and also highlights those reactions that would be essential regardless of the chosen media condition (unless new transporters are added).
2. We recommend you **do not** perform iterative gap filling in this step (do not use the --iterativegf flag on minimal media).

You can define your own media conditions with which to perform gap filling by creating a Media object in your workspace using **fba-addmedia**. However, the KBase also has about 700 default media conditions saved in the workspace **KBaseMedia**. To fill gaps in the model and achieve growth on a specific media condition, *fill gaps on complete media first* (since that solution is a basis for all other media conditions) and then call **fba-gapfill** again and use these flags:

-m $MEDIA_NAME --mediaws KBaseMedia

**11. Checking growth on minimal media**

To run FBA to a **specific media** (with a Media object in a workspace) use the **-m** flag (and **--mediaws** if the media is not in your current workspace) to the **fba-runfba** command. The default media for KBase are found in the KBaseMedia workspace so use the following to run FBA on one of those media conditions (you can also create your own media and put it in your workspace, in which case use that workspace instead with the—mediaws argument):

$ fba-runfba --fba $FBA_ID --media $MEDIA_NAME --mediaws KbaseMedia \

$MINIMAL_GAPFILLED_MODEL_NAME

If you imported your media condition using **fba-addmedia** you can specify that media (and your workspace name) instead.

**12. Importing phenotype data**

Note that we have already done this for the phenotype data used in the manuscript and saved them in the KBasePhenotypeDatasets workspace. If you only want to use those, you can skip this step. However, you will need to do this to do simulations of your own phenotype data.

The phenotype data is imported from a tab-delimited table with the following headers (the headers must be exactly the same as this, but can be in any order):

- **media** - Name of the Media object containing the media for which the phenotype was measured
- **mediaws** - Workspace in which the Media object above is located (often KBaseMedia, or your own workspace)
- **growth** - 1 for Grows, 0 for Does Not Grow
- **geneko** - OPTIONAL. If specified, it is a semicolon-delimited list of gene knockouts. The gene IDs must match the IDs from your original genome source (e.g. SEED IDs in form fig|#.#.peg.# where each # is some number).
- **addtlcpd** - OPTIONAL. If specified, it is a semicolon-delimited list of compounds added to the specified Media condition before measuring the phenotype (use it for example to record the effects of making small changes to media and testing effects on growth).

Example: You did knockouts of "fig|83333.1.peg.1", "*fig|83333.1.peg.2*" and "*fig|83333.1.peg.3*" separately and tried to grow your organism on Carbon-D-Glucose. The ∆*fig|83333.1.peg.1* strain grew but the ∆*fig|83333.1.peg.2* and ∆*fig|83333.1.peg.1* strains did not. The input file would then look like this: (note that the separator between each field is a tab, including in the header).

media mediaws growth geneko

Carbon-D-Glucose KBaseMedia 1 fig|83333.1.peg.1

Carbon-D-Glucose KBaseMedia 0 fig|83333.1.peg.2

Carbon-D-Glucose KbaseMedia 0 fig|83333.1.peg.3

Example 2: You tried a triple knockout of these three genes and it did not grow. You can add a line like this to the above file to account for this:

Carbon-D-Glucose KbaseMedia 0 fig|83333.1.peg.1;fig|83333.1.peg.2;fig|83333.1.peg.3

Note that it is possible to import other IDs such as locus tags into a genome file and then use them in the phenotype table. Doing so is outside the scope of this tutorial but see **fba-importtranslation** for details.

Once you have set up this table, import it into IRIS using its file import capability and then run:

$ fba-importpheno $GENOME_ID $PHENOTYPE_FILE --phenoid $PHENOTYPE_SET_ID

**13. Phenotype simulations**

Run phenotype simulations using the **fba-simpheno** command. In order to run this you will need a phenotype set. Several phenotype sets for the organisms we discussed in the manuscript are available in the KBasePhenotypeDatasets workspace. Otherwise you will need to import your phenotype data as a PhenotypeSet object (**step 12**) first.

We recommend that for knockout simulations you first try to run FBA on the media in which the knockouts were performed (**step 11**). If you get a 0 growth rate, you should run gap filling to that media so that you don't get all negative growth predictions. If you do this, don’t use iterative gap filling, but use either likelihood or parsimony-based gap fill algorithms depending on what you have been using to get this far. Once you have a growing model just use **fba-simpheno** to simulate the phenotype.

$ fba-simpheno $MODEL_NAME --phenows KBasePhenotypeDatasets $PHENOTYPE_SET_ID \

--phenosimid $OUTPUT_SIMULATIONS

**--phenosimid** should be specified if you want to give the resulting phenotype simulation set a specific name (recommended). You can then take a look at that object and identify the correct and incorrect growth predictions relative to the available phenotype data.

For biolog simulations you should perform gap filling to achieve growth on a minimal media (such as Carbon-D-Glucose) before running simulations. You should also specify **--alltransporters** in the **fba-simpheno** command so that transporters are automatically added for all compounds in all tested growth conditions before simulation is done. This is necessary because transporters are the hardest to get right, and it is quite likely that the model will be missing transporters that, if present, would allow the cell to grow with the rest of what's in the network. The final command to use becomes:

$ fba-simpheno $MODEL_NAME --phenows KBasePhenotypeDatasets $PHENOTYPE_SET_ID \

-- phenosimid $OUTPUT_SIMULATIONS --alltransporters
