# Supplementary material for: Likelihood-Based Gene Annotations for Gap Filling and Quality Assessment in Genome-Scale Metabolic Models
Source: PLoS Comput Biol. 2014 Oct 16;10(10):e1003882. doi: 10.1371/journal.pcbi.1003882 (PMC4199484; doi:10.1371/journal.pcbi.1003882)
Supplement: Text S3 — Use of the KBase client API. Documentation for use of the client API provided as Software S1. (DOC) [file pcbi.1003882.s009.doc]

**Description of the KBASE Client API for Likelihood-based gap filling workflows**

This tutorial describes how to build metabolic models using the four workflows that we have described in the manuscript. There are actually two ways to run this workflow:

1. Using the **Client API**, or
2. Using the **Web-based command line interface**

This tutorial describes a script that uses the client API to perform the workflows. Please see the other tutorial file (Supplemental file 2) for a detailed description of the workflows themselves.

**Obtaining a Globus Online account**

You will need to create a Globus Online account and a workspace (see Supplemental file 2 for details) before running the workflow script. These will be used for logging into the KBase and to authenticate that you have permission to save to and read from your workspace.

Do not use the same password as used in other websites in case the password is echoed and logged somewhere.

**Creating a workspace**

You will need to create a workspace before running the workflow script. See Text S2 for details on how to do that.

**Obtaining the KBase client API**

First you will need to have the following packages installed:

- Python, version 2.7.x
  - The following built-in packages are used by client libraries: urllib2, httplib, urlparse, random, base64, ConfigParser, json
  - The following built-in packages are used by the workflow script: argparse, subprocess, traceback, sys, time, operator
- Python packages (get using apt-get or pip or whatever you have to install it):
  - httplib2

For example in Ubuntu do the following to get httplib2

$ sudo apt-get install python-httplib2

Second, download the "Software S1" (ZIP file) provided with this manuscript. Unzip it with the program of your choice.

Finally, add the extracted directory (the one that contains the "biokbase" directory) to your PYTHONPATH or alternatively run "python setup.py install" to install it to your system path (depending on your system you might need to run this with "sudo" to avoid permission errors).

**Running the workflow script**

The workflow script has the following syntax:

usage: Workflow [-h] [--password PASSWORD] [--createws CREATEWS]

[--genome_source SOURCE] [--parsimony] [--likelihood]

[--iterative] [--iterative-likelihood]

[--num-solutions NUMSOLUTIONS] [--knockout KNOCKOUT]

[--knockoutws KNOCKOUTWS] [--biologdata BIOLOG]

[--biologdataws BIOLOGWS] [--positiveTransportersOnly]

[--maxtime MAXTIME] [--ws-url WSURL] [--job-url JOBURL]

[--fba-url FBAURL] [--pa-url PAURL] [--ujsurl UJSURL]

[--transporter-penalty TRANSPORTERPENALTY]

[--direction-penalty DIRECTIONPENALTY]

genome workspace username

The required arguments are a genome ID (by default coming from the PubSEED, [http://pubseed.theseed.org](http://pubseed.theseed.org/)), a workspace and a username. The script asks for a password interactively if it is not provided on the command line. **The first time you run it** if you don't already have a workspace, use --createws to create one.

**Example usage**

Run parsimony-based gap filling workflow on E coli K12 (which has genome ID 83333.1) and use the phenotype dataset '83333.1-essentiality' found in KBasePhenotypeDatasets to simulate the resulting model.

$ python Workflow.py --parsimony --knockout 83333.1-essentiality

--knockoutws KBasePhenotypeDatasets 83333.1 MYWORKSPACE MYUSERNAME

Run likelihood-based gap filling workflow with the same organism and phenotype data

$ python Workflow.py --parsimony --knockout 83333.1-essentiality

--knockoutws KBasePhenotypeDatasets 83333.1 MYWORKSPACE MYUSERNAME

**Alternative genome sources**

You can specify that you want your genome to come from the KBase central store instead of the PubSEED by specifying this using --genome_source . Supported values include "kbase" or "seed".

**The four workflows**

You can specify which workflow to run using the following flags:

- --parsimony: Run the targeted parsimony-based gap fill workflow (activate biomass only)
- --prob : Run the targeted likelihood-based gap fill workflow (activate biomass only)
- --iterative: Run the iterative parsimony-based gap fill workflow
- --iterativeprob: Run the iterative likelihood-based gap fill workflow

You can also specify that you want to run the biolog or knockout lethality phenotype predictions after gap filling using:

- --biologdata: For biolog data
- --knockout: For knockout data

For biolog data you have the option of fitting to it by adding transporters only for growth-positive substrates using --positiveTransportersOnly. By default, transporters are added for ALL media in the biolog data before running simulations.

If a gap fill job fails due to lack of time you can increase the time limit using --maxtime. The argument is provided in units of seconds.

**Note**: The servers are prone to random failures (504s etc). If you restart the workflow script with the same arguments as were used before the error, it will start back up where it left off. It is recommended to check the results of the last step first to see if an object is present and if results are in it - you might need to delete the object (using ws-delete) before starting the workflow again. Also check to see if your job is still running - the workflow might have just failed while checking to see if your job was done. If this happens, wait for the job to finish and then restart the workflow using the same arguments as you used before (except don't try to create the workspace a second time).

**Specifying where your data is**

All data used in Workflow.py is extracted from and saved in workspaces. By default the Workflow.py looks for and saves everything in same workspace as you specify on the command line.

Alternatives to the default can be specified as follows:

- --knockoutws: This specifies where knockout data is located, if you are running that part of the workflow.
- --biologdataws: This specifies where biolog data is located, if you are running that part of the workflow.

In order to run the Workflow, you must have write permissions in the main workspace (-w) and read permission in the knockout/biolog workspaces. I suggest using IRIS (see other tutorial) to create the workspace and manage permissions.

**Specifying what servers to use**

Every server is specified by a URL. If the URL ever changes you will need to specify the new URL using the URL arguments (or go in and change the default):

- --ws-url : Workspace service URL
- --job-url: URL for the service that handles FBA jobs (gap filling and reaction sensitivity)
- --fba-url : FBA Modeling service URL
- --pa-url: Probabilistic Annotation service URL
- --ujs-url: User and Job Service URL

**Specifying gap fill parameters**

The penalties for unfavorable reversibility changes and for adding transport reactions can be adjusted from their defaults of 12 and 25 (respectively) by using the --direction-penalty and --transporter-penalty flags, respectively.
