# Supplementary material for: Likelihood-Based Gene Annotations for Gap Filling and Quality Assessment in Genome-Scale Metabolic Models
Source: PLoS Comput Biol. 2014 Oct 16;10(10):e1003882. doi: 10.1371/journal.pcbi.1003882 (PMC4199484; doi:10.1371/journal.pcbi.1003882)
Supplement: Text S4 — Directions for use of the provided VM. How to set up and run your own services to run the code. (DOC) [file pcbi.1003882.s010.doc]

**Directions for using the Probabilistic Annotation and FBA Modeling Image**

Note: The password for the kbase user on the VM is "kbase".

These directions are used to access the server code for probabilistic annotation, FBA modeling and their dependencies on the provided virtual machine. Like all distributions the virtual machine and the code within is provided strictly on an AS IS basis.

The virtual machine file will always have a file format "likelihood-gapfill-[date].ova". It will always be located in the following FTP directory: <ftp://dtn.chicago.kbase.us/assets/> . It will be periodically updated with bugfixes and the version number incremented accordingly.

Note that the directions in this document **only** pertain to setting up your own server. Once it is set up you run the commands in the same way as you would using the web-based command line interface (see **Text S2** for details).

**Setting up the VM in VirtualBox**

The Probabilistic and FBA modeling Image is provided as a .vdi file, which is a Hard Drive file with expandable space that is natively supported in VirtualBox. You will need VirtualBox to use it.

In VirtualBox go to

File -> Import appliance…


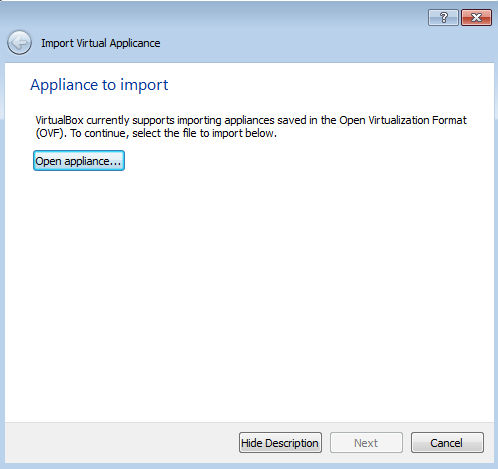


Click "Open appliance…" then click through the next couple dialogs until your machine is created (note - importing the appliance can take about 5-10 minutes).

Once your machine is created you might want to give it more than one core. To do that go to "settings" and click on "system". Then go to "processor" and select the number of cores you want to assign to the virtual machine while it is running.

**
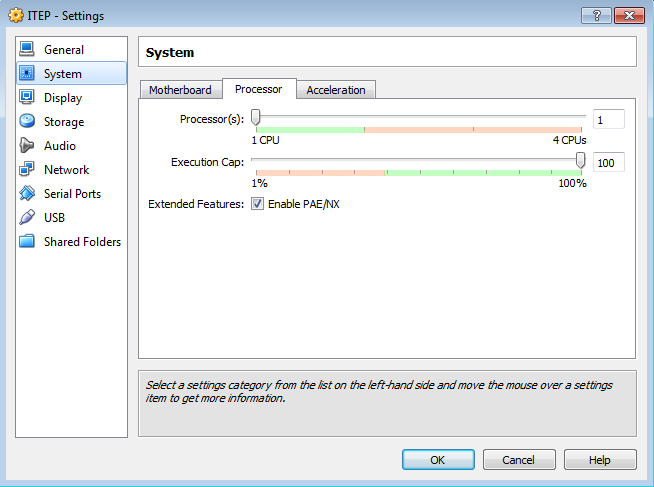
**

Note that you should probably leave assign at most one or two less than the number of processors available on your host system.

If you have extra memory available you might want to allocate more of that as well (using the "motherboard" tab), 512 MB won't cut it for some jobs especially if you want to run gap filling.


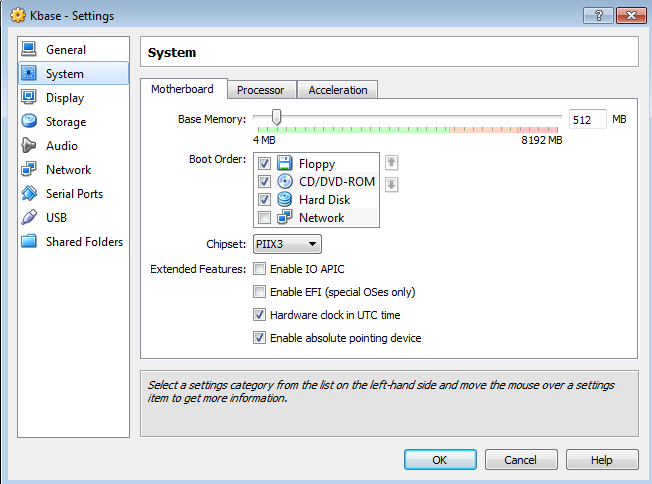


**Logging in**

You have to be root to log in successfully (I don't know why).

$ sudo bash

The password is kbase (no caps)

After doing this log into your KBase account using kbase-login:

$ kbase-login [your_username]

Type your password when prompted.

**Setting up paths**

To set up paths for service dependencies run the following command.

$ source /kb/deployment/user-env.sh

**Setting number of threads for probabilistic annotation (optional)**

If you set more than one core for your VM while setting it up, you can set up probabilistic annotation to use more than one core at once. To do this, run the following:

$ emacs -nw /kb/deployment/deployment.cfg

Change "blast_threads" to an integer that is less than or equal to the number of cores you assigned to the VM. Save by typing "CTRL+X" then "CTRL+S". Leave emacs by typing "CTRL+X" then "CTRL+C"

**IMPORTANT:** The other parameters here can be modified too. However, to preserve the integrity of the service we recommend **only** changing the following three parameters if you so choose:

dilution_percent #[Percent above maximum score to keep genes in the GPR]

pseudo_count #[The pseudocount parameter that limits likelihoods of genes with very few similar genes]

blast_threads #[Number of threads to use for BLASTP as part of likelihood calculation]

Any changes to the config file must be done before starting the services (or you will have to stop and restart the service for the changes to take effect).

**Starting the services**

To run code locally on the VM you need to first start the services that run the code:

$ /kb/deployment/services/fbaModelServices/start_service

$ /kb/deployment/services/probabilistic_annotation/start_service

**IMPORTANT:** Make sure you check the following two files for errors with starting the services (after running the above commands) before trying to run anything else.

/kb/deployment/services/fbaModelServices/error.log

/kb/deployment/services/probabilistic_annotation/error.log

You can check whether the services are running (they take about a minute to start up) using:

$ curl [http://localhost:7036](http://localhost:7036/) #fbaModelServices

$ curl [http://localhost:7073](http://localhost:7073/) #probabilistic_annotation

They should tell you "HTTP get not allowed" or "No JSON object could be decoded". If you get a 500 or 502 error or similar, something went wrong with the startup and the error log should tell you. If it just hangs the server is probably taking its time starting up.

**Setting up URLs and running things on the VM or some other machine**

The URL you set for the FBA and probabilistic annotation services determines what machine is used to run those commands. The URLs for the production KBase services are the following:

FBA modeling: https://kbase.us/services/KBaseFBAModeling

Probabilistic annotation: http://kbase.us/services/probabilistic_annotation

However, the VM is set up to run the services locally assuming that is the intended use case. The URLs are as follows:

FBA modeling: "http://localhost:7036"

Probabilistic annotation: "[http://localhost:7073](http://localhost:7073/)"

To change the URL to production or to some other machine you have set up, use 'fba-url' and 'pa-url':

$ fba-url <FBA_modeling_service_URL>

$ pa-url <PA_modeling_service_URL>

Note that gap fill jobs themselves will NOT run on your machine by default - they will be put on the job queue for the SEED. You can still pull them off the job queue by using

$ fba-runjob [jobid]

and they will run on the VM.
